# Exploiting signal transduction pathways for cancer therapy: insights from natural products in preclinical models

**DOI:** 10.3389/fphar.2026.1804164

**Published:** 2026-05-20

**Authors:** Sarad Pawar Naik Bukke, Balakrishna Vuyyala, Chandrashekar Thalluri, Ananda Kumar Chettupalli, Neha Gupta, Muhammad Ali Abdullah Almoyad, Safa A. Abdalla, Vinod Kumar Yata, Alrazi Eisa Shogar, Bayapa Reddy Narapureddy, Zohre Eftekhari, Patrick Maduabuchi Aja, Tadele Mekuriya Yadesa

**Affiliations:** 1 Department of Pharmaceutics and Pharmaceutical Technology, Kampala International University, Kampala, Uganda; 2 Department of Pharmacology, Gurunanak College of Pharmacy, Gurunanak Institutions Technical Campus, Hyderabad, Telangana, India; 3 Faculty of Pharmaceutical Science, Assam Down Town University (AdtU), Guwahati, Assam, India; 4 Department of Pharmaceutical Technology, School of Health and Medical Sciences, Adamas University, Barbaria, West Bengal, India; 5 Department of Pharmaceutics, Aligarh College of Pharmacy, Aligarh, Uttar Pradesh, India; 6 Department of Basic Medical Sciences, College of Applied Medical Sciences, Khamis Mushyt, King Khalid University, Abha, Saudi Arabia; 7 Department of Clinical Pharmacy and Pharmacy Practice, Kampala International University, Kampala, Uganda; 8 Department of Biotechnology, School of Allied and Healthcare Sciences, Malla Reddy University, Hyderabad, Telangana, India; 9 Department of Public Health, College of Applied Medical Sciences, King Khalid University, Abha, Saudi Arabia; 10 Department of Biotechnology, Pasteur Institute of Iran (IPI), Tehran Province, Tehran, Iran; 11 Department of Biochemistry, Faculty of Biomedical Sciences, Kampala International University, Kampala, Uganda

**Keywords:** cancer metabolism, mutations, natural products, preclinical models, signal transduction pathways

## Abstract

Genetic or epigenetic changes that lead to abnormal development of signalling through cellular pathways such as MAPK, PI3K/AKT/mTOR, Wnt/β-catenin play an important role in driving cancer. These signalling pathways control how cells grow, divide and die (apoptosis). Thus, the continued activation of these pathways represents a characteristic of oncogenesis as well as a suitable target for treatment. This review will highlight how natural products have been shown to modify the molecular pathways involved with these signalling cascades in preclinical cancer models. Data derived from an array of preclinical studies showcase how the bioactive phytochemicals that comprise these products are implemented in the form of targeting cancer cells through specific inhibition of signalling pathways, induction of apoptosis, inhibition of tumour growth and alteration of oncogenic crosstalk. Despite the significant body of evidence, there are still several limitations that exist in regards to variability between studies in terms of the methods used for validation of the mechanism by which these compounds function, differences in their bioavailability and overall robustness of those studies. Using a pathway-centered understanding of the mechanisms by which natural compounds exert their therapeutic effects will support the development of targeted phytopharmaceutical approaches and promote the integration of these therapies into evidence-based oncology practices.

## Graphical Abstract

**Figure:**
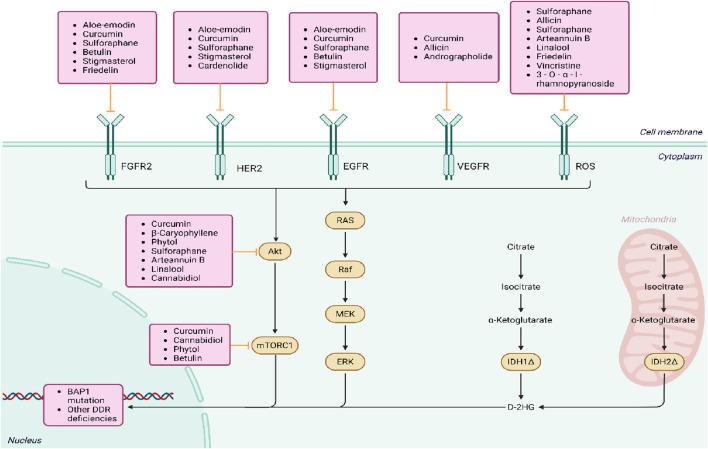

## 1 Introduction

Cancer, characterized by uncontrolled proliferation of abnormal cells, can arise in any bodily tissue or organ (Ruggeri and Cancer, 2021). These cells form tumours, infiltrate nearby tissues, and metastasize, leading to severe complications. Also known as a neoplasm or malignant tumour, effective treatment often involves a combination of surgery, chemotherapy, radiation therapy, immunotherapy, or targeted therapy, tailored to the specific type and stage of the disease. Quick identification and intervention are important to enhance prognosis and decrease death rates related to cancer.

Normally, human cells replicate through cell division, producing cells on demand. Cell death or deterioration typically undergo programmed cell death to facilitate replacement by new cells. However, disruptions in this orderly process can lead to uncontrolled proliferation of abnormal or damaged cells. A tumour is a growth of aberrant tissue formed during cell division, either excessively or failing to undergo programmed cell death (Slika et al., 2023).

### 1.1 Epidemiology

The aim of cancer’s epidemiology is to look at how the cancer is distributed and its determinants in all populations so that one is able to get insight into how much of a burden the disease causes, what risk factors contribute to the disease, and how to decrease the incidence of the above disease (Hamada et al., 2019; Hughes, 2008). Global cancer data from various sources such as the (World Health Organization, 2019; Huber et al., 2022) Global Burden of Disease study show many geographic menstrual cycles of cancer and major sex differences in cancer include lung cancer, (different from other cancers due to both) the differences in incidence and mortality due to how patients respond to treatment, and, (different from other cancers due to both) the differences in geographical sex differences in the incidence of lung cancer compared to men.

Major causes of cancer related deaths worldwide are lung cancer, liver cancer and stomach cancer. Of all the cancers that are caused by tobacco, (mostly from smoking), the most common cancer in women is breast cancer and the most common cancer in men is prostate cancer. Increasingly, colorectal cancers are being diagnosed as a result of changing lifestyle choices, contributing to the world’s cancer (Siegel et al., 2021). Epidemiological trends for cancers are important in order to establish targeted prevention, early detection and public health interventions.

**FIGURE 1:**
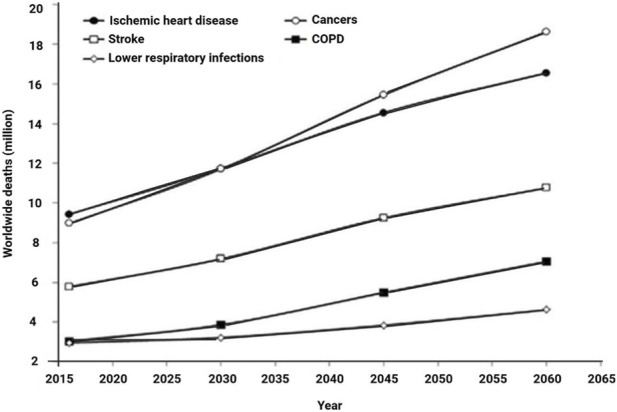
Projected epidemiological trends of the five major causes of mortality between 2016 and 2060. COPD, chronic obstructive pulmonary disease (2019).

The World Health Organization’s (WHO) latest update in 2016 presented the top twenty sources of illness burden, quantified as cause-specific disability-adjusted life years (DALYs) (Figure 1).

According to the 2019 global cancer burden estimates, there are significant differences in both incidence and mortality for cancers depending on geographic areas and sex (Mattiuzzi and Lippi, 2019). The GLOBOCAN 2018 data estimated approximately 12.7 million new cancer cases and 7.6 million cancer deaths around the world, demonstrating the very high global burden that cancer represents. The incidence rates of cancer vary tremendously across population groups, with the potential for an incidence difference by up to six times for men compared to women across different registries. Some of the differences in the incidence of cancer are a result of differences in the distribution of risk factors, socio-economic conditions, and access to healthcare among different populations. It should be noted that certain cancers are more prevalent among different genders and in different regions, emphasizing the importance of having customized cancer prevention and control strategies that take the influences of each specific cancer on the burden of cancer in each population into consideration. The list of the types of cancers that are most common can be found in Table 1 and is based on GLOBOCAN 2018 data provided by the WHO Global Cancer Observatory.

**TABLE 1:** List of the most frequent cancers.

Cancer	Incidence (million)					Risk 0–74 years (%)		
	Male	Female	Total	Ratio	Age standardized	Total	Men	Women
All cancers	9.456	8.623	18.079	1.10	197.9	20.2	22.41	18.25
Lungs	1.369	0.725	2.094	1.89	22.5	2.75	3.8	1.77
Breast	-	2.088	2.088	-	46.3	-	-	5.03
Prostate	1.276	​	1.276	-	29.3	-	3.73	-
Colon	0.576	0.521	1.097	1.11	11.5	1.31	1.51	1.12
Stomach	0.684	0.350	1.034	1.95	11.1	1.31	1.87	0.79
Liver	0.597	0.245	0.841	2.44	9.3	1.08	1.61	0.57
Rectum	0.430	0.274	0.704	1.57	7.7	0.91	1.2	0.63
Esophagus	0.400	0.172	0.572	2.32	6.3	0.78	1.13	0.43
Cervix	-	0.570	0.570	-	13.1	-	-	1.36
Thyroid	0.131	0.436	0.567	0.30	6.7	0.68	0.33	1.03

### 1.2 Cancer patterns vary globally

In men, lung cancer is most common in Eastern Europe and Asia; prostate cancer in the Americas, Australia, and Europe; liver cancer in West Africa; oesophageal cancer in East Africa; and bladder cancer in Egypt. In women, breast cancer leads in most regions, while cervical cancer is prevalent in Sub-Saharan Africa, India, and parts of Latin America. Liver cancer is most common in Mongolia and Vietnam, and lung cancer in China and North Korea.

This review highlights incidence and mortality trends, excluding Kaposi sarcoma and bladder cancer for brevity (Lin et al., 2021; Ahmed et al., 2013).

#### 1.2.1 Causes of cancer

The primary catalyst for cancer is the alteration of genes responsible for regulating cell growth, division, and apoptosis. Various factors contribute to these genetic changes, including:

- Inherited genetic predispositions
- Formation of cells mistakes
- Natural pollutants cause DNA damage.

While our bodies have mechanisms to eliminate cells with damaged DNA and prevent cancer initiation, this defence weakens with age, making the elderly more vulnerable to cancer. Once a cell undergoes malignant transformation, its genetic profile continues to evolve, resulting in a heterogeneous population of cells within a single cancerous mass (Ahmedin et al., 2010). Uncontrolled cell division and metastasis to other tissues constitute cancer (Table 2).

**TABLE 2:** Difference between cancer cells and normal cells (Eldridge, 2023).

Category	Normal cells	Cancerous cells
Growth	Follow a controlled pattern	Unchecked expansion
Interaction	Take up information from neighbouring cells	Ignore impulses sent by neighbouring cells
Survival or demise of cells	Old or broken cells are either restored or replaced	Repairing or replacing cells is not possible
sticky or dispersive	remain in designated areas	Are capable of moving independently and all around the body
First impression	The same appearance when examined under a microscope	Different sizes, darker centre with the microscope
Developing into an adult	Attain adulthood	Avoid maturation
Impersonating a pathogen	Can be targeted and eliminated	Hide and grow without interruption
Purpose	Perform designated tasks	Neglect assigned tasks
The flow of blood	Blood vessels grow to feed normal growth and aid in repairs	Unstoppable blood vessels fuel tumours

## 2 Causes and risk factors

Cancer occurs when cells lose their natural control over growth, division, and programmed cell death (Ahmedin et al., 2010). Cancer develops as a result of one or more of the following genetic alterations, either inherited (passed down through families) or acquired (due to exposure to potential carcinogens (cancer-causing substances), such as tobacco smoke and exposure to ultraviolet and ionizing (e.g., x-ray) radiation, and exposure to certain industrial chemicals (Ahmedin et al., 2010). In addition, hormonal imbalance has been implicated in the development of many hormone-dependent cancers (e.g., breast and prostate). Other infectious agents are responsible for considerable numbers of diagnosed cancers throughout the world, including human papillomavirus (HPV, cervical cancer), hepatitis B and C viruses (liver cancer), and *Helicobacter pylori* (gastric cancer, also known as stomach cancer) (Ahmedin et al., 2010). Chronic inflammation can also lead to cancer by causing long-term (chronic) cellular stress and instability of the DNA (Schauer and Linton, 2009; Thun et al., 2017) (the inability of the DNA in a given cell to produce normal proteins due to the inability of that cell to produce DNA). Although there are many modifiable risk factors (i.e., risk factors that can be altered in some way), smoking is the most important modifiable risk factor (i.e., the leading cause of preventable cancer deaths). Radiation exposure (whether from exposure to medical radiation (e.g., x-rays) or from exposure to environmental radiation) is another important modifiable risk factor that leads to cancer development. Moreover, individuals with weakened immune systems (e.g., those who have had a transplanted organ) are at increased risk for developing cancer because of their decreased ability to mount an immune response against cancer cells. Together, these factors emphasize the complex and preventable nature of many of the cancers described here (Mettler et al., 2008).

## 3 Lifestyle and age-related determinants

The relationship between dietary habits and cancer is less rigidly concluded in terms of its relationship to tobacco use because dietary patterns have many variables and it is hard to measure if someone has consumed that food for an extended period of time (Engels et al., 2011). Although a few specific nutrients have been found to provide protective effects, there is more difficulty in identifying these nutrients and how they promote carcinogenesis since there is not any appreciable way to determine if they have a causative effect in isolation (Freedman et al., 2011). On the contrary, obesity is well-established and is an increasingly prevalent modifiable cancer risk for numerous cancer types. Obesity is only one of the main factors associated with the development of malignant diseases through mechanisms such as chronic inflammation, dysfunction of hormones (including insulin), insulin resistance, and alterations in the signals from growth factors. Strong, direct causal relationships exist between obesity and postmenopausal breast cancer, colorectal cancer, pancreatic cancer, endometrial cancer, and kidney cancer, and esophageal cancer. Additionally, there is evidence that obesity (see increased body mass index) increases the likelihood of developing liver and kidney cancers. In comparison to obesity as a cancer risk factor, the relationship with smoking is more clear and established; moreover, smoking is an established causative risk factor for several types of cancer, and additional research has yet to confirm whether weight loss is likely to alleviate the risk of cancer associated with obesity (Bhaskaran et al., 2014).

### 3.1 Pathophysiology of cancer

**FIGURE 2:**
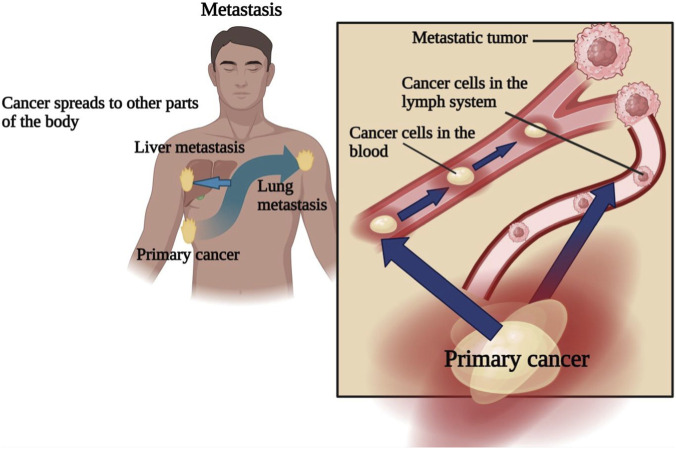
Schematic representation of cancer metastasis.

Malignant tumors develop from the abnormal proliferation and survival of cells resulting from genetic (mutational) and epigenetic (non-mutational) changes that allow for a breakdown of normal cell regulatory mechanisms. Tumorigenesis starts with individual cells that have accumulated mutations leading to abnormal proliferation. The process of tumorigenesis leads not only to a mass of tumor cells, but also to the accumulation of greater numbers of both genetic and epigenetic mutations, giving rise to new clones of tumor cells that all exhibit the same characteristics. The accumulation of genetic and epigenetic mutations confers advantages for the tumor cell population as a whole (increased growth, development of resistance to cell death, etc.) and the subpopulations of tumor cells become increasingly heterogeneous over time, with progressively more aggressive subpopulations dominating the overall tumor mass. Therefore, as a tumor develops, there are will be increases in the ability of cancer cells to metastasize, with complex multistep processes through which malignant cells dissociate from the main tumor mass and invade nearby organ structures, then enter the blood stream and lymphatic system, and ultimately establish secondary tumors in distant organs. Secondary tumors retain many of the molecular and histological characteristics of primary tumors, and are the primary cause of cancer-related death (Wolin and Colditz, 2008). Importantly, these secondary tumours retain the histological and molecular features of the primary cancer rather than adopting the characteristics of the organ in which they reside (Figure 2).

### 3.2 Development of cancer

**FIGURE 3:**
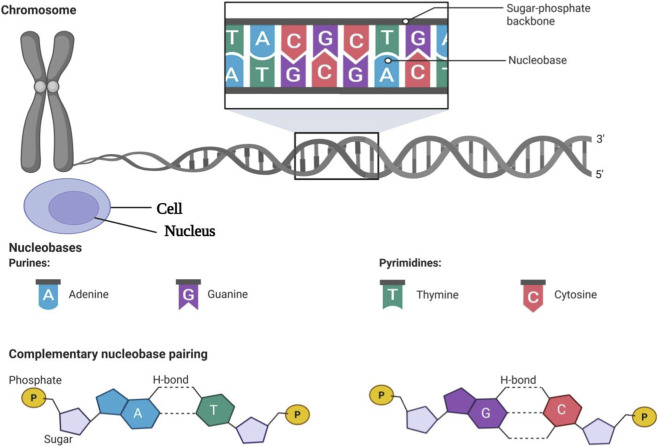
Structure and organization of DNA, complementary base pairing, and mutations.

The phrase “Cancer which has spread away from its primary site, to involve distant organs” is used to define tumours that develop NATURALLY in organ/tissue of their origin with regard to the above. For instance, if breast cancer were to “metastasise” into the lungs as a metastasis, those secondary lesion(s) will retain the same morphological and molecular features as the original breast tumor. Despite advances made through the use of new therapies, metastatic disease is to be treated with a predominantly palliative approach, although still focusing on the control of the neoplasia and providing symptomatic relief to the patient, since ALL metastases will be responsible for the majority of cancer-related death(s). At the molecular level, cancer arises from acquired genetic changes that lead to abnormal proliferation, aberrant cellular differentiation and/or aberrant regulation of apoptosis. Genetic changes (mutations) can occur due to inherited genetic factors, environmental carcinogens, and also chance events in the DNA replication process. In general, increased age correlates with more genetic mutations in the DNA of cells because the cell’s ability to repair DNA becomes impaired, and therefore, the immune system’s ability to “detect and destroy” (via a variety of modes) defective cells declines with age. The continuous accumulation of genetic mutations and resulting genomic instability ultimately generates the development of tumour heterogeneity, clonal evolution, progressive therapeutic resistance, and eventually the development of tumours that demonstrate increased clinical aggressiveness (Rompianesi et al., 2019) (Figure 3).

#### 3.2.1 Cell signalling pathways

**FIGURE 4:**
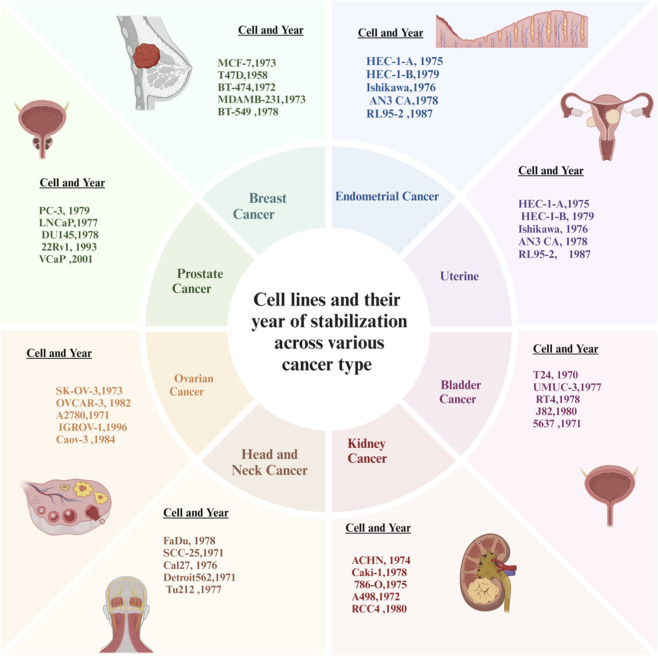
Cancer cell lines across tumour types and years.

Cancers are known as dysregulated or disorganized systems because of the role of impaired cell-to-cell signaling in the development and spread of cancer. In addition to promoting a sustained proliferation rate, dysregulated signaling is also involved in inducing genomic instability and providing the ability for cancer cells to evade programmed cell death (apoptosis). Manipulation of key cell-to-cell signaling pathways has been implicated as the mechanism by which dysregulated cell-to-cell signaling promotes malignant transformation, and thus provides important pharmacologic targets for therapy (Figure 4). The following perspectives address three key oncogenic signaling pathways (the mitogen-activated protein kinase (MAPK) pathway, phosphatidylinositol-3-kinase (PI3K)/AKT/mTOR cascade and Wnt/β-catenin pathway) and their associated biological functions, including proliferation, differentiation, and survival. Collectively, continued signaling through all three of these pathways represents elements of oncogenesis which have strong correlations to the process of cancer development and progression, and therefore are considered optimal strategies for anticancer therapies. The cancer development process is primarily associated with the alterations (mutations) of three major classes of genes. The first class of genes within this classification scheme is the proto-oncogenes, which are responsible for determining/control cell proliferation. Proto-oncogenes become oncogenes, when either mutated or expressed at levels higher than normal cells, and provide a stimulus that drives uncontrolled cell proliferation. The second class of genes comprises tumor suppressor genes; they are normally responsible for regulating/censoring the growth of cells, and thereby maintaining genomic stability. Tumor suppressor gene inactivity results in the loss of critical regulatory mechanisms for the growth of cells. The third class of genes consists of DNA repair genes; these genes allow for the correction/repair of damaged nucleic acid within the genome. When either mutated or expressed inappropriately, the loss of functional DNA repair genes results in further accumulation of genetic changes, including major chromosomal aberrations. Together, disruption of the three aforementioned gene classes can collectively promote the malignant transformation of normal cells to cancerous cells, thus driving overall cancer progression.

#### 3.2.2 Types of cancer

There are six primary kinds of cancer: carcinomas, sarcomas (both benign and malignant), lymphoma, leukemia, adenomas, and malignant germ cell cancers (Wytiaz et al., 2025; National Comprehensive Cancer Network, 2024). Cancer cell lines are critical non-animal (*in vitro*) models used to study tumor biology and test therapeutic agents (Sonali et al., 2021). They are derived from primary tumors and established to grow continuously in a controlled laboratory environment. Cancer cell lines provide an easily reproducible platform for the investigation of molecular mechanisms, drug responses, resistance pathways, and cytotoxic effects of drugs on cancer cells (Dai et al., 2017). In addition, there are hundreds of well-characterized cancer cell lines that have been established to represent different types of cancer and their biological subtypes (Lax, 2004; Skok et al., 2020; Terzic et al., 2021). The most commonly used animal models in breast cancer research include MCF-7, T47D, BT-474, and MDA-MB231, which allow researchers to study the effects of hormones on breast cancer development and treatment as well as how cancer spreads to different parts of the body. Multiple models are also available for the study of endometrial cancer, such as HEC-1-A, Ishikawa, and RL95-2, which allow for hormone-induced cancer development studies (McConkey et al., 2009; Knowles and Hurst, 2015). Bladder cancer cell lines (T24, UMUC-3, RT4), (Eisen et al., 2012; Hernandez et al., 2016), kidney cancer cell lines (786-O, Caki-1, ACHN), and head and neck squamous cell carcinoma cell lines (FaDu, SCC-25, Cal27) (Leemans et al., 2011; Stransky et al., 2011) are used to understand the genetic mutations associated with kidney and/or bladder cancer and how they spread to other parts of the body, as well as to evaluate how resistant cancers are to chemotherapy drugs. Likewise, two of the main ovarian cancer cell lines (SK-OV-3, OVCAR-3, A2780) are commonly used to develop and evaluate inhibitors of platinum-based chemotherapy resistance (Domcke et al., 2013; Hernandez et al., 2016). Finally, prostate cancer cell lines (LNCaP, PC-3, DU145, VCaP, 22Rv1) are used in both hormone-sensitive and hormone-resistant prostate cancer research. Overall, the use of these cell lines demonstrates the diverse molecular characteristics of cancer and their importance in the fields of cancer research and drug development [34, 35].

**FIGURE 5:**
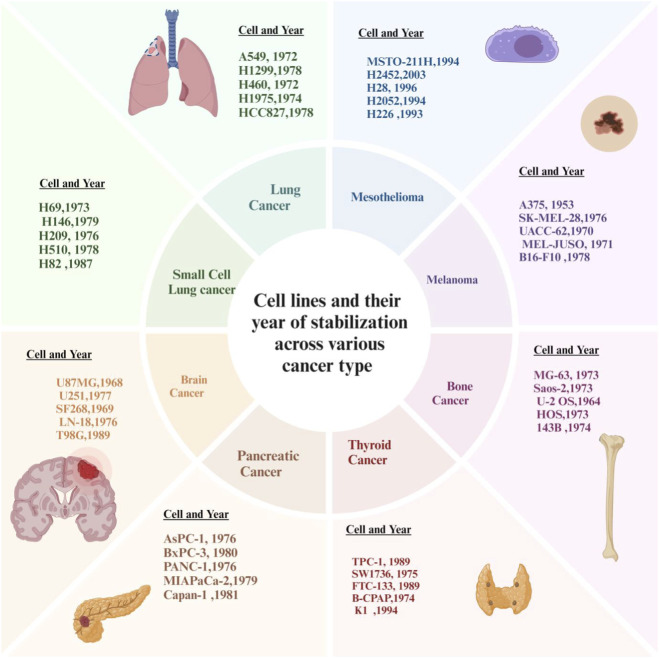
Year of establishment for selected cancer cell lines.

The molecular diversity of the main types of cancer is represented in these preclinical models by a variety of extensively characterized cancer cell lines (36). For example, NSCLC A549 and H460 (KRAS mutant) cell lines are becoming routinely used to study MAPK/ERK signalling (Gazdar et al., 2010), and they are also widely used as preclinical models in NSCLC of tumors with mutated KRAS; in contrast, H1975 and HCC827 cells are used to study EGFR-driven oncogenesis. Also, H1299 cells, which are devoid of functional p53, can be used for studies of tumor progression that are p53-independent. In the field of malignant pleural mesothelioma (MPM), models such as MSTO-211H, H2452, H28, H2052, and H226 provide opportunities to study tumor heterogeneity, drug resistance, invasion, and oncogenic signalling (Broeckx et al., 2018). In melanoma, A375 and SK-MEL-28 (BRAF mutant BRAF) cell lines are key for evaluating BRAF inhibitors (Hoek and Goding, 2010), while UACC-62 and MEL-JUSO cells provide opportunities to evaluate drug resistance and the response to immunotherapy. Figure 5 presents the years of establishment for selected cancer cell lines, illustrating the historical progression and development timeline of widely used experimental models in cancer research. B16-F10 is a highly useful mouse model when looking at both metastasis and the interactions between tumors and the immune system. Examples of pancreatic ductal adenocarcinomas include, but are not limited to, AsPC-1, BxPC-3, PANC-1, MIAPaCa-2, and Capan-1. Each represents some aspect of PDAC, including but not limited to, metastatic potential, chemoresistance, epithelial-to-mesenchymal transition (EMT), genetic alterations, and the interaction between tumor cells and stromal cells (Hidalgo, 2010). Osteosarcoma models include MG63, Saos-2, U-2 OS, HOS, and the highly metastatic 143B. Each of these cell lines represents different levels of differentiation and can be used as tools to evaluate the metastatic potential of osteosarcoma (Ottaviani and Jaffe, 2009; Currie et al., 2013; Dai et al., 2017). The following thyroid cancer models can be used to study RET/PTC rearrangements, BRAF mutations, RAS signaling pathways and aggressive anaplastic phenotypes; TPC-1, B-CPAP, K1, FTC-133, and SW1736 (Nikiforov, 2011; Saiselet et al., 2012; Coca-Pelaz et al., 2020). Glioblastoma models can be found in the following cell lines; U87MG, U251, LN-18, T98G and SF268. All five of these cell lines provide a good platform to test for EGFR/PTEN dysregulation, treatment resistance and p53 mutations (Vogelstein and Kinzler, 2004; Lathia et al., 2015). The 5 cell lines below (H69, H146, H209, H510, and H82) are examples of SCLC models that can be used to investigate neuroendocrine differentiation, loss of tumor suppressor function, mechanisms of apoptosis, and multiple drug resistance (Pietanza et al., 2015). As a whole, this wide variety of cancer cell lines can serve as powerful experimental systems for studying the oncogenic signalling pathways and mechanisms of therapeutic resistance to guide the discovery and development of targeted therapies for cancers.

**FIGURE 6:**
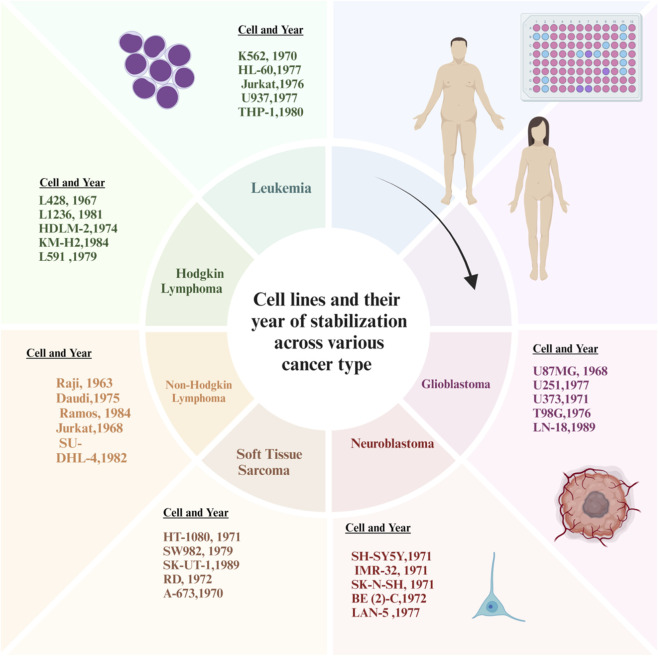
Cancer cell lines by type and year (hematologic and neural tumours).

K562, HL-60, Jurkat, U937, and THP-1 are some of the most prominent cell lines used in research in the area of leukaemia. K562 is used as a model of BCR-ABL–driven chronic myeloid leukaemia. K562 is also an important tool for the understanding and development of tyrosine kinase inhibitors. HL-60 is used to study differentiation therapy, especially how leukaemic cells respond to retinoic acid (Hozumi, 1985). Jurkat cells serve as important models of T-cell leukaemia. Researchers use Jurkat cells to investigate T-cell receptor signal transduction and apoptosis. U937 and THP-1 are both monocytic cell lines that are commonly used as model systems to study cytokine signalling, differentiation, and the inflammatory process. Figure 6 depicts cancer cell lines categorized by tumour type and year of establishment, with emphasis on hematologic and neural malignancies, highlighting their development trends over time. The cell lines SH-SY5Y, IMR-32, SK-N-SH, BE (2)-C and LAN-5, are widely used in neuroblastoma research and represent various stages of differentiation, as well as MYCN amplification status. Therefore, these cell lines are useful for the study of aggressiveness in tumours, as well as mechanisms of resistance to treatment and the development of more effective targeted therapies (Davidoff, 2012). U87MG, U251, U373, T98G, and LN-18 are all important models of glioblastoma and are primarily used to study EGFR signalling, p53 alteration, genomic instability, and therapeutic resistance in glioblastoma (Haque et al., 2011). The following soft tissue sarcomas are represented by the following cell lines: HT-1080 (fibrosarcoma), SW982 (synovial sarcoma), SK-UT-1 (leiomyosarcoma), RD (rhabdomyosarcoma), A-673 (Ewing’s sarcoma with EWS-FLI1 fusion). These various histological subtypes of the above-mentioned sarcomas will be utilized to support a wide variety of studies dealing with invasion, proliferation, and development of targeted therapies. The following Hodgkin lymphoma (HL) cell lines will be used to conduct studies of Reed-Sternberg cell biology, NF-κB signaling, cytokine regulation, and tumor-microenvironmental interactions: L428, L1236, HDLM-2, KM-H2, and L591 (Riggi and Stamenkovic, 2007). For non-Hodgkin lymphoma (NHL), Raji cells (Burkitt’s lymphoma), Daudi cells (T-cell lymphoma), Ramos cells (diffuse large B-cell lymphoma), Jurkat cells (T-cell lymphoma) and SU-DHL-4 cells represent the above three NHL subtypes. Investigations with the above NHL will be used to study EBV-directed oncogenesis, B-cell receptor signaling, apoptosis, and targeted therapeutic approaches (Küppers, 2009; Bienz et al., 2020). Both the hematological and solid tumor cell line models identified above are critical tools for conducting mechanistic research and translational development of targeted therapies across all types of malignancies (Cang et al., 2012).

Researchers studying colorectal cancer often use several different types of cell lines. The most commonly used colorectal cancer cell lines include HCT-116, HT-29, SW620, DLD-1, and Caco-2. HCT-116 and DLD-1 are considered to be two of the best models available for the study of the oncogenic signalling pathways and mechanism of resistance due to their KRAS-mutated background. HT-29, which bears mutations in BRAF and p53, is most commonly used to evaluate aberrant proliferation and resistance to apoptosis. SW620 is another good model of cancer progression and metastasis because it is derived from a metastatic lymph node and possesses both KRAS and APC mutations. Conversely, Caco-2 is a cell line that undergoes spontaneous differentiation to become enterocyte-like. This cell line is extremely valuable for studying intestinal physiology, epithelial permeability, and drug absorption. Collectively, these cell lines provide excellent models for studying the heterogeneity of colorectal cancers, and this heterogeneity can drive translational research.

Cholangiocarcinoma is a rare cancer of the bile ducts. Researchers studying cholangiocarcinoma also have several excellent models to study this cancer and advance the understanding of bile duct cancer. HuCCT1 and TFK-1 cell lines are both commonly used in studies designed to evaluate the proliferation, genetic alterations and signalling pathways of cholangiocarcinoma. Sk-ChA-1 cells are primarily used to evaluate the metastatic potential of cholangiocarcinoma tumours and to study targeted therapies, while KMBC cells are used primarily to study interactions between oncogenes and tumour-suppressor genes. SG231 cells are used to evaluate drug resistance and combination treatment regimens. These models continue to support the advancement of mechanistic and therapeutic research in cholangiocarcinoma (Jarnagin and Shoup, 2004).

Research into the therapy for adrenocortical carcinoma extensively utilizes NCI-H295, its steroidogenic subline NCI-H295R, SW13, MCF-7/ADR, and SK-Hep-1 as cell culture models. Both NCI-H295 and NCI-H295R are principal models for steroid biosynthesis, endocrine regulation, and resistance to therapies. SW13 is an additional model for studying aggressive behaviours in tumours and response to therapy. MCF-7/ADR is utilised in comparative studies of multidrug resistance and SK-Hep-1 is a metastasis or control model. Collectively, these cell culture models can be used to study the biology of adrenal cancer tumours, their regulation by hormones, and their resistance to therapy (Sidhu et al., 2003).

For gallbladder carcinoma research, key *in vitro* cell lines used as experimental models include TGBC2TKB, GBC-SD, NOZ, OCUG-1, and GB-d1. Gallbladder carcinoma cell lines are routinely used to study tumour growth, invasive properties, genetic changes, multiple drug resistance, and other aspects of drug efficacy. When combined, these gallbladder carcinoma cell lines are excellent models to conduct mechanistic studies and support the development of new therapies to treat gallbladder carcinoma (García et al., 2020).

Five cell lines used as research models for understanding gastric carcinoma include MKN-45, MKN-28, AGS, SNU-1, and KATO III. While MKN-45 and SNU-1 are used to model poorly differentiated tumours and chemoresistance, MKN-28 models differentiated gastric cancer (Sirica, 2005; Banales et al., 2020). AGS is an important model for studying Wnt/β-catenin signaling and has been used to screen for potential therapies. KATO III is used to model signet-ring-cell carcinoma, a rare subtype of gastric cancer that has a very aggressive course. All of these models continue to be essential to the study of gastric carcinogenesis and the response of gastric carcinomas to therapies (Tan and Yeoh, 2015; Garattini et al., 2017).

There are also 5 cell lines used to model esophageal cancer. OE19 and OE33 model adenocarcinomas of the esophagus, while KYSE-150, KYSE-30, and TE-1 model squamous cell carcinomas of the esophagus. Researchers use these cell lines extensively to study molecular mechanisms, tumor progression, and therapy evaluation. They represent a strong foundation for future studies of targeted interventions (Lin et al., 2018).

HepG2, Huh-7, PLC/PRF/5, Hep3B, and HepG2/C3A serve as the basis for hepatocellular carcinoma research. Both HepG2 and HepG2/C3A cell lines have been widely used for the study of hepatic metabolism and the toxicity of different drugs. Researchers studying the development of hepatocellular carcinoma due to Hepatitis C (HCV) use the Huh-7 cell line, while the Hep3B cell line is used for the study of Hepatitis B (HBV)-associated liver cancer. Researchers studying alpha-fetoprotein (AFP) and AFP-related biomarkers frequently utilize the PLC/PRF/5 cell line as an aid in their research. All of these cell lines are vital recording systems for the investigation of liver tumors and for the development of new liver cancer therapies (Goto et al., 2020).

These models are indispensable tools in contemporary cancer research, providing reproducible and physiologically relevant systems for investigating tumour biology, mechanisms of drug response, and the evaluation of novel therapeutic strategies. Once successfully established, cancer cell lines can be propagated *in vitro* over extended periods while maintaining key genetic and phenotypic characteristics of the original tumour. This long-term culture capacity eliminates the need for repeated cell isolation from patient samples and facilitates standardized experimentation across laboratories. Furthermore, cell lines can be cryopreserved for long-term storage, ensuring a renewable and consistent resource that supports ongoing and future investigations into cancer pathogenesis and treatment (Masters, 2000).

## 4 Plants reported to have anticancer activity

Various herbs and plants have been found to contain natural compounds that help to prevent cancer. These compounds, known as bioactive phytochemicals, include alkaloids, flavonoids, terpenoids, and phenolics, among others (Cang et al., 2012; Kumar et al., 2016). They can inhibit the growth of cancer cells, promote their death (apoptosis), reduce the formation of new blood vessels (angiogenesis), and alter their signaling pathways. Some examples of plants with anticancer compounds include: Catharanthus roseus (vincristine and vinblastine), Taxus brevifolia (paclitaxel), Camptotheca acuminata (camptothecin), Podophyllum peltatum (podophyllotoxin), Curcuma longa (curcumin), and Withania somnifera (withanolides). Tinospora cordifolia, Nigella sativa, and Andrographis paniculata also have cytotoxic and immunomodulatory properties. These plants remain significant sources for discovering new natural anticancer medications (Table 3).

**TABLE 3:** Plants reported to exhibit anticancer activity.

S No.	Plant name	Family	Part used	Extract/Isolated	Ref.
1	*Aloe vera*	Aloaceae	Whole plant	Ethanolic	Karpagam et al. (2019)
2	*Abutilon pannosum*	Malvaceae	Aerial branches	Ethanolic, chloroform, butanol and methanol	Al-zharani et al. (2023)
3	*Abelmoschus esculentus*	Malvaceae	Fresh flowers	Ethanol and ethyl acetate fraction	Solomon et al. (2016)
4	*Amaranthus viridis*	Amaranthacea	Leaves and stem	Ethanol	Sarker et al. (2019)
5	*Acalypha californica*	Euphorbiaceae	Aerial parts (stems, leaves,flowers and fruits)	Methanol	Rascón-Valenzuela et al. (2015)
6	*Artabotrys hexapetalus*	Euphorbiaceae	Leaves	Ethanol	Sun et al. (2024)
7	*Andrographis paniculata*	Acanthaceae	Leaf	Water, ethanol and acetone	Shukla et al. (2015)
8	*Apium graveolens*	Apiaceae	Seed	Aqueous, ethanolic and hexane	Hartono et al. (2024)
9	*Allium sativum*	Liliaceae	Fresh bulb	Ethanol, n- hexene and water	Bhat (2020)
10	*Artemisia annua*	Asteraceae	Stem and leaves	Ethanol and water	Ryu et al. (2011)
11	*Alstonia yunnanensis*	Apocynaceae	Diels roots	Methanol	Lai et al. (2022)
12	*Anacardium occidentale*	Anacardiaceae	Leaves	Hydroethanolic	Konan et al. (2012)
13	*Alstonia boonei*	Apocynaceae	Stem and bark	n-hexane and methanol	Balogun et al. (2016)
14	*Bauhinia purpurea*	Fabaceae	Leaves	Aqueous chloroform	Zakaria et al. (2011)
15	*Berberis vulgairs*	Berberidaceae	Fruit	Alcoholic and aqueous	Hoshyar et al. (2016)
16	*Bauhinia tomentosa*	Fabaceae	Root	Ethanol	Tuama and Mohammed (2019)
17	*Boerhaavia diffusa*	Nyctaginaceae	Leaves	Ethanol	Das et al. (2023)
18	*Betula utilis*	Betulaceae	Barks	Ethyl acetate, chloroform, methanol and water	Khan et al. (2019)
19	*Beberis aristata*	Berberidaceae	Bark and stems	Water, methanolic and ethanolic	Borah et al. (2020)
20	*Bidens pilosa*	Asteraceae	Aerial, whole plant and leaves	n-hexane, ethyl acetate, methanol, ethanol, hydro alcoholic, acetone, chloroform and water	Sundararajan et al. (2006)
21	*Brassica oleracea*	Brassicaceae	Leaves, stem,seeds	Dichloromethane ethanol, methanol and water	Li et al. (2023)
22	*Brassica napus*	Brassicaceae	roots, Leaves	Hexane ethanol, methanol, or water	Jan et al. (2018)
23	*Bambusa*	Poaceae	Leaves	Dichloromethane ethanol, methanol, or water	Patel and Mehta (2021)
24	*Bauhinia*	Fabaceae	Leaves	Ethanol, Methanol, Water and Dicholoromethane	Patil et al. (2023)
25	*Cascabela thevetia*	Apocynaceae	Leaves, Seeds, and Flowers	Ethanol, Methanol and Dicholoromethane	Sowjanya et al. (2013)
26	*Cordyline frutico*	Asparagacea	Leaves, roots and stems	Ethanol, Methanol and Dicholoromethane	Tematio Fouedjou et al. (2023)
27	*Curcuma longa*	Zingiberaceae	Rhizome	Ethanol, Methanol, hexane and acetone	Kuttan et al. (1985)
28	*Coriandrum sativum*	Apiaceae	Whole plant	Ethanol, Methanol and Dicholoromethane	Tang et al. (2013)
29	*Cinnamonus cassia*	Lauraceae	Bark	Ethanol, Methanol, Dicholoromethane and hexane	Ariaee-Nasab et al. (2014)
30	*Cassia tora*	Fabaceae	Leaf	Ethanol, Methanol, Dicholoromethane and hexane	Rao (2013)
31	*Cassia fistula*	Fabaceae	Fruit	Ethanol, Methanol, Dicholoromethane and hexane	Boontha et al. (2023)
32	*Caesapinia pulcherrima*	Fabaceae	Whole plant	Ethanol, Methanol and Dicholoromethane	Deepika et al. (2020)
33	*Coleus amoboinicus*	Lamiaceae	Leaves, stem and flower	Ethanol, Methanol, Dicholoromethane and hexane	Tewari et al. (2012)
34	*Cycas circinalis*	Cycadaceae	Coralloid roots	Ethanol, Methanol, Dicholoromethane and hexane	Moawad et al. (2010)
35	*Cannbis sativa*	Cannabaceae	Leaves, stem,flower	Olive oil, Butane, Hexane, Carbon Dioxide and ethanol	Tematio Fouedjou et al. (2023)
36	*Catharanthus roseus*	Apocyananceae	Dried whole plant	Acetone, ethanol and Methanol	Rates (2001)
37	*Cenntella asiatica*	Apiaceae	Whole plant	Ethanol, Methanol, Dicholoromethane and hexane	Babu et al. (1995)
38	*Dianthus chinensis*	Caryophyiiales	Roots, leaves, and stems	Ethanol, Methanol, Dicholoromethane and hexane	Nho et al. (2012)
39	*Dypsis lutescens*	Arecaceae	Leaves	Ethanol, Methanol, Dicholoromethane and hexane	Akshaya et al. (2022)
40	*Daphniphyllum*	Daphniphyllaceae	Leaves	Ethanol	Yang et al. (2021)
41	*Deacaena reflexa*	Asparagaceae	Leaves	n-butanol fraction	Ghalloo et al. (2022)
42	*Dictamnusdasycarpus*	Rutaceae	Leaves and Stems, fruit	*Methanolic*	Qin et al. (2021)
43	*Eugenia caryophyllata*	Myrtaceae	Seeds	*Clove essential* oil	Amrati et al. (2021)
44	*Ficus benjamina*	Moracea	Leaves	*Methanol and chloroform*	Imran et al. (2014)
45	*Garcinia indica*	Guttiferae	Fruit	Ethanol, methanol, and water	Jayakar et al. (2021)
46	*Glycyrrhiza glabra*	Leguminoseae	Extract of plant	Water and ethanolic	Hamad et al. (2020)
47	*Gardenia gummifera*	Rubiaceae	Flower	*Methanolic*	Vinaykumar et al. (2020)
48	*Jatropha integerrima*	Euphorbiaceae	Trunks	*Methanolic*	Mohammed et al. (2021)
49	*Momordica charantia*	Cucurbitaceae	Leaves, stem bark, and fruit	Hydroalcoholic, (Triterpenoids, phenolic acids and flavonoids)	Ray et al. (2010)
50	*Melissa*	Lamiaceae	Leaves	Methanolic, ethanolic, and aqueous extracts	Saraydin et al. (2012)
51	*Mentha arvensis*	Lamiaceae	Whole plant	*Methanolic extracts* (Menthol and rosmarinic acid)	Jerard et al. (2020)
52	*Pyrostegia venusta*	Bignoniaceae	Roots, leaves, and stems	*Methanolic,* ethyl acetate and Heptane *extract* (Octacosane)	Figueiredo et al. (2014)
53	*Pulmaria rubra*	Apocynaceae	Leaves	*Methanolic* (Phenolics and flavonoids)	Elgizawy et al. (2024)
54	*Phyllanthus emblica*	Phyllanthaceae	(fruits, seeds, leaves, roots, bark, and flowers)	*Methanolic* (Polyphenols, tannins, and flavonoids)	Saini et al. (2022)
55	*Saussurea lappa clarke*	Compositae	Driedroots	*Methanol and* n-butanol (Costunolides)	Ansari et al. (2021)
56	*Solanum melogena*	Solanaceae	Peal	*Methanolic* (Solanine, chaconine, Solamargine and tomatina)	Fekry et al. (2019)
57	*Samanea saman*	Fabaceae	Leaves	Methanol and hexane	Vinodhini and Devi Rajeswari (2018)
58	*Tabernaemontana divaricata*	Apocynaceae	Leaves, Roots, and Bark	Ethanol and chloroformic extract	Kumar and Selvakumar (2015)
59	*Tabebuia impetiginosa*	Bignoniaceae	Stem bark	Methanol	Son et al. (2006)
60	*Typhonium flagelliforme*	Araceae	Juice of whole plant	Methanol, ethyl acetate and hexane	Lai et al. (2010)
61	*Tinospora cordifolia*	Menispermceae	Whole herbs	Methanol	Ali and Dixit (2013)
62	*Tecoma stans*	Bignoniaceae	Leaves, flower and Bark	Methanol	Narayanan et al. (2023)

The medicinal potential of plants is based on their biochemical make-up and their role in the ecosystem or as a part of nature’s therapeutic benefit to humans. Secondary metabolites produced by specialized biosynthetic pathways are called metameters and they are mainly produced for defensive and adaptive purposes, rather than for growth-related functions. The chemical structures of secondary metabolites are related to and/or influence biological activity. For example, alkaloids have been demonstrated to interact with nucleic acids and enzymes to produce anticancer and antimicrobial activities. In addition, flavonoids and phenolics are known to have antioxidant and signaling properties, while terpenoids and glycosides demonstrate anti-inflammatory and cytotoxic characteristics. Knowledge of the chemical structures and biosynthetic pathways of these compounds is important to explaining the therapeutic efficacy of medicinal plants and advancing the development of natural-product-based drugs (Figure 8).

Many different plants contain bioactive/active compounds that have the potential to produce therapeutic effects through targeted molecular interactions. Phytochemicals can interact with important cellular components such as proteins, enzymes, and receptors to impact signaling pathways that regulate cellular functions (e.g., proliferation, apoptosis, inflammation, and metastasis); these biochemical mechanisms represent the basis of many plant-based medicines.

Some examples of how particular plant-based/derived compounds produce therapeutic effects are provided. Vincristine and vinblastine (Catharanthus roseus) inhibit the assembly of microtubules by binding to tubulin, whereas paclitaxel (Taxus brevifolia) stabilizes microtubules and induces G2/M cell cycle arrest. Camptothecin (Camptotheca acuminata) disrupts DNA replication by inhibiting topoisomerase I. Curcumin (Curcuma longa) modulates the NF-κB, STAT3, COX-2 and PI3K/Akt pathways, exhibiting both anti-inflammatory and pro-apoptotic activities. Withaferin A (Withania somnifera) targets vimentin and NF-κB signalling pathways, thereby contributing to the anticancer activity of this compound (Table 4).

**TABLE 4:** Plant-derived bioactive compounds exert their pharmacological effects by interacting with specific receptors, enzymes, or signalling molecules that regulate key physiological and pathological processes.

S. No	Plant name	Identified compounds	Site of binding to receptors/modulators	Ref.
1	*Aloe vera*	Aloe emodin	i. Probably by suppressing CCND2 transcript levels ii. Activation of Caspase-6	Sanders et al. (2017)
2	Abutilon pannosum	Phytol	Activation of Caspase-7	Al-zharani et al. (2023)
3	Abelmoschus esculentus	β-caryophyllene	The expression of caspase 3 and 7 and the circadian genes clock and Bmal1 using RT-PCR.	Abdel-Razek et al. (2023)
4	*Acalypha californica*	β-sitosterol	Activates caspases 3, 8 and 9	Rascón-Valenzuela et al. (2015)
5	Andrographis paniculata	Andrographolide	Affect signalling pathways, including the JAK/STAT pathway, Wnt/β-catenin pathway, and mTOR signalling pathway	Kumar et al. (2004)
6	*Allium sativum* L	Allicin	Enhancing p38 expression and cleaved caspase 3	El-Saber Batiha et al. (2020)
7	*Artemisia annua*	Arteannuin B	Caspase 3 activation	Lang et al. (2019)
8	*Alstonia yunnanensis*	Acetoxytabernosine	Expression of apoptosis-related proteins (Caspase9, Caspase3, and Parp-1)	Lai et al. (2022)
9	*Anacardium occidentale*	Camptothecin	Binds to the topoisomerase I and DNA complex	Pham et al. (2023)
10	*Berberis vulgaris*	Berberine	i. Acting on Ras-MAPK and PI3K/Akt signal cascade pathways ii. Inhibition of MAPK/mTOR/p70S6K and Akt signalling pathways	Hoshyar et al. (2016)
11	Betula utilis	Betulin	Activation of caspase-8 and caspase-9 Ber	Mishra et al. (2016)
12	*Beberis aristata*	Berberine	Significant binding affinity with EGFR	Pai et al. (2012)
13	*Bidens pilosa*	Stigmasterol	Expression of Bax and Caspase-3	Shen et al. (2018)
14	*Brassica oleracea*	Sulforaphane	Regulating p53 and Bax (proapoptotic) proteins, as well as caspase-3	Devi and Thangam (2012)
15	*Brassica napus*	Glucosinolate	Reducing the expression of Bcl2 and activating p-p53, caspase-3, caspase-8, and caspase-9	Mandrich and Caputo (2020)
16	*Bambusa*	Triterpenoids	Downregulating Bcl-2 and upregulating Bax, caspase-9, and caspase-3	Pant and Husen (2025)
17	*Bauhinia*	3,5,7,3′,5′-pentahydroxyflavanonol-3-O-α-l-rhamnopyranoside	BaP induced X57BL/6	Yuenyongsawad et al. (2013)
18	*Cascabela thevetia*	Cardenolide	Na/K-ATPase, which is a promising drug target in cancer	Long et al. (2024)
19	*Curcuma longa*	Curcumin	Suppression of HER2/neu and EGFR activity	Zhou et al. (2011)
20	*Coriandrum sativum*	Linalool	Inhibition of *HDAC activity*	Iwasaki et al. (2016)
21	*Cinnamonus cassia*	Cinnamaldehyde	Decreased expression of MMP-2, and downregulation of Her-2 expression	Khedkar and Ahmad Khan (2023)
22	*Cassia tora*	Friedelin	Raf and, MEK are molecular targets	Joshi et al. (2023)
23	*Cannbis sativa*	Cannabidiol	Bind to CB_1_, CB_2_, and other G protein-coupled receptors	Kovalchuk and Kovalchuk (2020)
24	*Catharanthus roseus*	Vincristine and Vinblastine	Bind to the microtubular proteins of the mitotic spindle	Mohammadgholi et al. (2013)
25	*Cenntella asiatica*	Asiatic Acid	Elevated expression of microtubule-associated protein 1 light chain 3 (LC3) and decreased the expression of p62	Wu et al. (2017)
26	*Dianthus chinensis*	Quercetin	Activation of Caspase-3/7, −8, and −9	Nonpunya et al. (2014)
27	*Dypsis lutescens*	Isovitexin	Wnt/β-catenin signalling pathway suppression and nuclear factor-κB protein expression inhibition	Tan et al. (2026)
28	*Eugenia caryophyllata*	Eugenol	Promotes apoptosis by altering Bcl-2 family proteins via the mitochondrial route	Zari et al. (2021)
29	*Garcinia indica*	Garcinol	Activate p-mTOR, p-GSK-3β and p-PI3K/Akt signalling pathway	Lim et al. (2021)
30	*Glycyrrhiza glabra*	Glycyrrhetinic acid	Activates the caspase cascade and mitochondrial apoptotic pathway	Tuli et al. (2022)
31	*Momordica charantia*	Plumericin	Inhibited proliferation, and induced G2/M cell cycle arrest and apoptosis. It significantly decreased expressions of COX 2 and VEGF in the cells	Sur and B. Ray (2023)
32	*Mentha arvensis*	Menthol	modulating the MAPK and PI3K/Akt pathways	Zhao et al. (2023)
33	*Phyllanthus emblica*	Quercetin	Activation of EGFR also triggers downstream MAPK–ERK intracellular signalling pathway	Samatiwat et al. (2021)
34	*Saussurea lappa Clarke*	Sesquiterpenes	Altering the activity of nuclear factor kappa (NF-kB), preventing lipid peroxidation, and delaying the generation of reactive oxygen and nitrogen species (ROS&RNS)	Kumar and Pundir (2022)
35	*Samanea saman*	Lupeol	Inhibits LMP1-induced NF-κB activation and reduces NF-κB-dependent LCL viability	Pitchai et al. (2014)
36	*Tabebuia impetiginosa*	Lapachol	i. Reduced Bcl-2 expression, increased Bax expression, and activated caspases 3 and 9 ii. Expression of ERK1/2 MAPK	Zhang et al. (2020)
37	*Typhonium flagelliforme*	Pheophorbide	Increased, but anti-apoptotic Bcl-2 protein levels decreased	Yu et al. (2024)
38	*Tinospora cordifolia*	Berberine	Cyclin A2, which can bind to CDK2 and is essential for cell proliferation, is encoded by the gene CCNA2	Palmieri et al. (2019)
39	*Tecoma stans*	3,5-dicaffeoylquinic acid	Ability to interact with BCL2 (B-Cell Lymphoma 2) and VEGFR2 (Vascular Endothelial Growth Factor Receptor 2)	Narayanan et al. (2023)

Phytochemicals (plant-derived natural products) can all be thought of as synergistically interacting with numerous molecular receptors (including structural proteins, enzymes, etc.), and thus their ability to act as multi-target agents may provide ample opportunity for their own future use in molecule-based drug design either as naturally-occurring compounds themselves or by novel synthetic chemistries (taking their active pharmacophores and creating synthetically-derived versions) but even more so, as chemical scaffolds upon which many fine-tuned, second/third generation therapeutics could be developed.

Phytochemicals exert their effects by binding to specific molecular receptor sites within the body (such as certain types of receptor proteins, certain types of enzymes, certain types of signal transduction proteins, etc.) and by doing so modulate the activity of the various intracellular pathways associated with specific disease processes, such as cancer, inflammation, oxidative damage, and infection. Therefore, an understanding of how and where these phytochemicals work within the body is critical in understanding their mechanisms of action and in helping with the discovery of new drugs.

Some examples of phytochemicals include: curcumin (Curcuma longa): inhibits NF-kB pathway, Scondary pathway, Akt pathway; resveratrol (Vitis vinifera): activates SIRT1 and estrogen receptors; EGCG (green tea, Camellia sinensis): inhibits angiogenesis through activation of laminin receptor and vascular endothelial growth factor receptor-2 (VEGFR2); withaferin A (Withania somnifera): inhibits NF-kB (the intracellular signalling pathway activated by many proinflammatory cytokines) and activates vimentin; quercetin: modulates PI3K/Akt, MAPK, and p53 pathways; berberine (Berberis vulgaris): binds to DNA and topoisomerases; paclitaxel (Taxus brevifolia): stabilizes -tubulin; camptothecin (Camptotheca acuminata): inhibits topoisomerase I; vincristine and vinblastine (Catharanthus roseus): inhibit microtubule polymerization; and thymoquinone (Nigella sativa): activates p53 and NF-kB pathways.

Together, these compounds illustrate the potential for synergistic (multi-target) effects of naturally-derived phytochemical scaffolds for the development of next-generation (safer and more effective) therapeutics.

As illustrated in Table 4, there are numerous phytochemicals that primarily affect only a limited number (1–3) of signaling pathways, the majority of which contain a significant number of important pathways that regulate apoptosis and the PI3K/AKT/MAPK cascades (Masters, 2000; Mathieu et al., 2019). Therefore, it is evident that these two (2) signaling axes represent a significant area for potential therapeutic intervention in cancer treatment, thereby providing evidence for the concept of ‘pathways converging’. A complete table of all natural substances that impact the induction of autophagy and apoptosis is provided in the summary table, as are their potential modes of action, experimental paradigms and molecular targets.

Table 5 shows the effects of various naturally occurring substances on the autophagy and apoptosis pathways in colorectal cancer, which may have therapeutic implications.

**TABLE 5:** Summary of natural compounds targeting autophagy and apoptosis pathways in cancer.

Natural compound	Target pathway(s)	Experimental model	Cancer type	Key findings	Clinical status
Curcumin	AMPK/mTOR, Beclin-1	*In vitro*, *in vivo*	Colorectal cancer	Induces autophagy and apoptosis via mTOR inhibition	Clinical trials
Clinical trials	SIRT1, AMPK	*In vitro*	Colon cancer	Promotes autophagy and suppresses tumor growth	Preclinical
Quercetin	PI3K/Akt/mTOR	*In vitro*, *in vivo*	CRC	Enhances apoptosis and autophagy	Preclinical
EGCG	MAPK, Bcl-2	*In vitro*	Colon cancer	Downregulates Bcl-2 induces apoptosis	Preclinical
Berberine	AMPK activation	*In vitro*, *in vivo*	CRC	Induces autophagic cell death	Preclinical

## 5 Treatment

A patient with cancer will require a management plan that integrates different specialties and that is tailored for the patient based on tumour type and stage of progression as well as molecular makeup and condition of the patient. The major treatment forms are surgery, chemotherapy, radiation therapy, immunotherapy, targeted therapy, hormone therapy and stem cell transplant plus supportive care (palliative care). Clinically, many treatment forms are used together to maximize effectiveness, minimize recurrence and improve long-term survival (Nall, 2024).

## 6 Innovative approaches

The liquid biopsy is a technique that allows for the detection of CTCs, ctDNA, and other cancer-related biomarkers without fully removing the tumor sample. With liquid biopsies, it is possible to monitor genetic mutations within the tumor as well as how the tumor responds to treatment in real-time, and it may provide an alternative option to a traditional tissue biopsy (Shegekar et al., 2023).

Precision medicine aims to provide patients with individualized treatment options through the use of genomic sequencing and biomarker profiling, which identifies specific mutations and allows for the identification of therapeutic targets. By using precision medicine, patients are more likely to receive effective treatments while also suffering from fewer side effects compared with traditional medication therapies (Shegekar et al., 2023).

**FIGURE 7:**
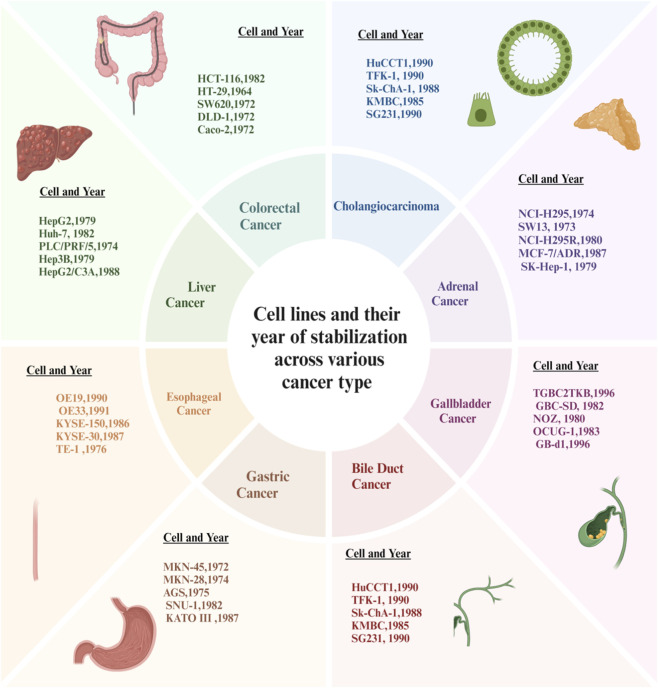
Cell lines originate from a range of biological sources, including normal tissues, immortalized cells, and tumour specimens the latter forming what are specifically known as cancer cell lines.

Cryoablation is a localized technique that uses either liquid nitrogen or argon gas to create extreme cold temperatures (causing tumor necrosis) to treat certain types of malignancies in the liver, kidney, prostate, skin, etc., by using a minimally invasive technique (Tsunoda et al., 2021). Figure 7 shows that cell lines originate from diverse biological sources, including normal tissues, immortalized cells, and tumour specimens (cancer cell lines).

**FIGURE 8:**
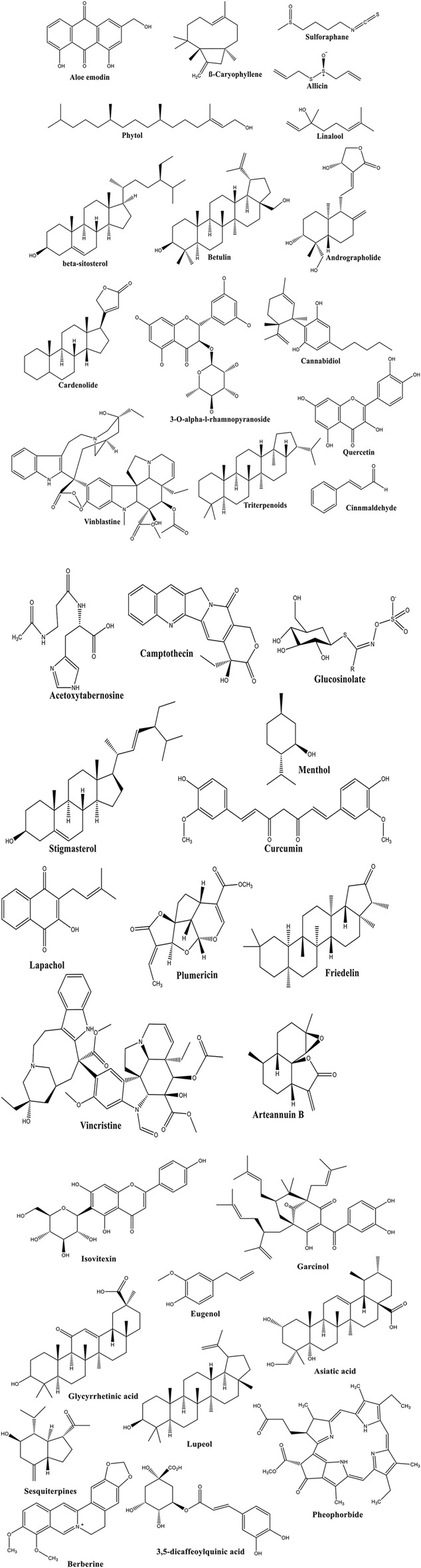
Plant-derived compounds and their binding sites or modulators.

Nanotechnology has the potential to revolutionize drug delivery systems by utilizing engineered nanosized particles that will allow for targeted drug delivery and improved imaging techniques. Engineered nanocarriers also help to improve tumor-specific accumulation of therapeutic agents while minimizing systemic toxicity. AI Uses sophisticated algorithms to evaluate imaging, genomic, and clinical data in order to develop more accurate diagnostics and optimize outcome prediction, diagnosis, and creation of personalized treatment plans. PDT Uses a combination of a photosensitizer agent, generation of reactive oxygen species through light and oxygen exposure, and tumor cell death via specific destruction, to treat superficial cancers (Agostinis et al., 2011). Combination Therapy Incorporation of many different treatment modalities (such as chemotherapy, immunotherapy, radiation therapy and targeted drug therapies) to work synergistically, to reduce resistance to individual modalities, and to provide the most optimal outcome for cancer treatment. Together, these new and innovative methods are revolutionizing the field of precision oncology and expanding the treatment options available through contemporary cancer treatment.

## 7 Drug conjugates with antibodies: a revolutionary strategy for targeted cancer treatment

There has been tremendous advancement in cancer treatment over the last century, especially through the use of biologic therapies that increase the precision and targeting of cancer treatments. Classic chemotherapy is designed to work against rapidly dividing cells, but it does not target cancer cells specifically and is damaging to normal tissues and also causes systemic toxicity.

Biologic therapies that have become advanced, targetable therapies are known as antibody–drug conjugates (ADCs). An ADC is a drug made up of an antibody that detects and attaches to specific tumour associated antigens or receptors. This specificity allows ADCs to deliver a highly toxic cytotoxic drug only to those cells that have been recognised by the antibody and the drug attached to it. Therefore, each ADC is designed to deliver the cytotoxic drug only to cancer cells and not to normal cells.

Monoclonal antibodies work well in the treatment of cancers, however, they have been poorly penetrable to solid tumours; and this is where ADCs come in. The ADC technology provides a more effective way to deliver the cytotoxic drug with greater accuracy and targetability. Incorporating both immunology and pharmacology techniques together in the development of ADCs provides significant advancement in precision oncology, resulting in lower side effects and greater efficacy (Lu et al., 2016).

## 8 Recent advancements in tumour-targeted therapies

Recent advances in tumor-targeted therapy have addressed the shortcomings of conventional chemotherapy by enhancing selectivity and reducing systemic toxicity. Antibody–Drug Conjugates (ADCs) are one of the most significant advancements in cancer treatment.

ADCs consist of three major components: a monoclonal antibody that selectively binds to tumor-associated antigens to allow for the selective internalization and intracellular release of a highly potent cytotoxic payload (active pharmaceutical ingredient) through a specialized chemical linker. This method allows for the maximization of antitumor efficacy while minimizing toxicity on normal tissues and is an example of precision medicine (Lu et al., 2016).

The manufacture of ADCs is a very complex and highly regulated activity that requires the precise synthesis and conjugation of the antibody, linker, and payload, followed by extensive purification to remove any free drug or unconjugated antibody. It is important to maintain a consistent drug-to-antibody ratio (DAR) when manufacturing ADCs, as it is critical for their safety and therapeutic efficacy (Lu et al., 2016; Lambert and Morris, 2017; Patel et al., 2023).

Antigen binding to ADCs results in multiple ways of achieving their goals through various mechanisms. Typical ways ADCs bind to antigens are through cysteine-based or lysine-based methods of linking and deliver their active components (payloads) through various routes. The most common payloads linked to ADCs include auristatins, pyrrolobenzodiazepines (PBDs) and irinotecan. Because of their ability to bind and deliver payloads through different routes, and because they have been shown to exhibit a broad range of activities, ADCs are advancing into a number of areas in pre-clinical and clinical testing, and are likely to be an important group of targeted therapies for the next-generation of cancer treatments (Fu et al., 2022).

## 9 Improved targeted cancer treatment: from immune-based strategies to antibody–drug conjugates

**FIGURE 9:**
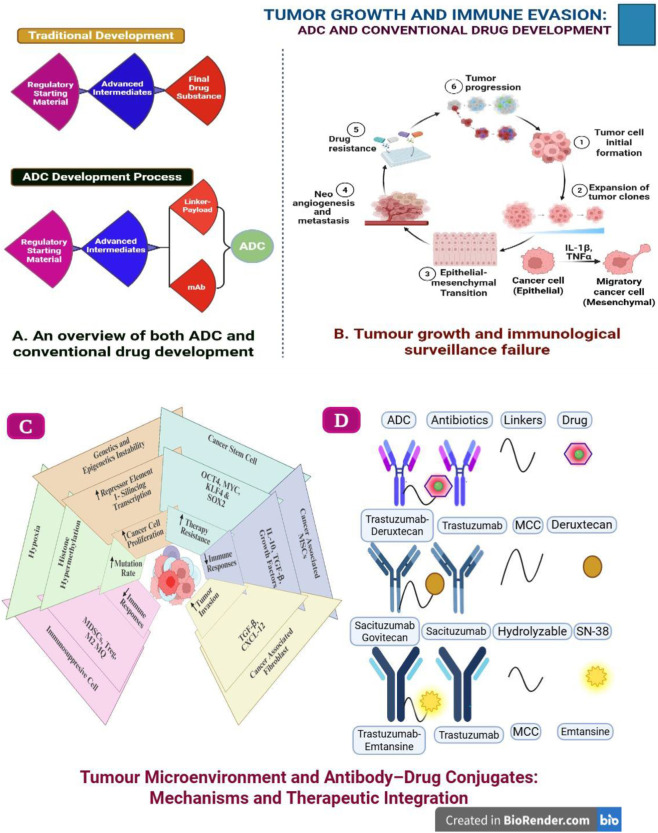
**(A)** Schematic comparison of conventional drug development and antibody–drug conjugate (ADC) development processes, highlighting key components such as monoclonal antibody (mAb), linker, and payload. **(B)** Illustration of tumor growth dynamics and immune evasion, depicting stages from tumor initiation to metastasis and drug resistance. **(C)** The tumour microenvironment’s cells’ properties that support tumour growth are altered by various immune regulatory cell types, hypoxia, mutations, epigenetic changes in the tumour tissue environment, and alterations brought on by malnutrition. **(D)** Comparison of T-DM1, T-DXd, and sacituzumab govitecan components in antibody–drug conjugates.

Tumour progression is frequently a result of a lack of effective immunity from the immune system. This lack of effective immunity allows tumours to develop mechanisms to avoid T-cell mediated immune responses. Immunotherapy aims to restore or increase the immune systems’ ability to recognize and kill malignant cells (Sfanos et al., 2018). Prostate cancer is considered immunologically active because of the presence of many tumour-infiltrating lymphocytes and the identification of numerous tumour-associated antigens within the prostate cancer micro-environment; therefore; it is believed to be a good candidate for treatment with immunotherapy. Presently, Sipuleucel-T is the only FDA approved immunologic therapeutic agent for the treatment of prostate cancer (Drake, 2010). The two main types of immunotherapeutic strategies are cell-based therapies, and antibody-based therapies, which both work to enhance the immune mediated anti-cancer effects of the immune system (Mellman et al., 2011). Figure 9 (A) Comparative schematic of conventional drug development and ADC design, emphasizing mAb–linker–payload architecture. (B) Overview of tumor progression and immune evasion, illustrating key stages from initiation to metastasis and therapeutic resistance.

## 10 Target cancer treatment and immunological evasion: from immune checkpoint regulation in prostate cancer to antibody–drug conjugates

Programmed cell death protein 1 (PD-1), expressed on activated T, B, and natural killer (NK) cells, interacts with its ligands PD-L1 and PD-L2 to attenuate T-cell activation and maintain immune tolerance (Quezada and Peggs, 2013; Xin et al., 2020). Malignant cells exploit PD-L1 expression to suppress effector T-cell activity and thereby evading immune surveillance (Figure 9C). PD-1 shares structural similarity with CD28 and CTLA-4, which are central regulators of T-cell activation and inhibition (Gevensleben et al., 2016). In prostate cancer, increased expression of PD-1 and PD-L1, together with PD-1 promoter hypermethylation, is associated with diminished PD-1 mRNA expression, elevated preoperative PSA, advanced tumour stage, and poorer recurrence-free survival, underscoring its role as an adverse prognostic indicator (Goltz et al., 2016).

## 11 Innovation in cancer targeted therapy: the growing significance of immunological modulations in solid tumours and antibody drug conjugates

The significance of incorporating ADCs into early BC management. By providing new choices for patients who have not responded well to conventional therapies, ADCs are revolutionizing cancer treatment, particularly in complex tumours like TNBC and HER2+/low. For several reasons, ADCs are crucial to the treatment of early-stage BC. These medicines’ capacity to deliver strong cytotoxic medications straight to cancer cells lowers systemic exposure and lessens adverse effects. Monoclonal antibodies (mAbs) that selectively target tumour-associated antigens are used to build of ADCs. A synergistic effect results when mAbs are conjugated with small-molecule chemotherapy agents through a chemical linker (Peters and Brown, 2015). The structures and modes of action of three FDA-approved medications sacituzumab govitecan (SG), fam-trastuzumab deruxtecan (TDXd), and trastuzumab emtansine (T-DM1) are shown in Figure 9D. The distinct structural characteristics of each of these ADCs contribute to their effectiveness. The ability of antibody–drug conjugates (ADCs) to continue to be helpful even after the disease has progressed is another important benefit in the early setting. This feature enables ADCs to more successfully target residual illness following neoadjuvant therapy, hence providing ongoing therapeutic benefits following progression in early lines of treatment (Peters and Brown, 2015).

## 12 Innovative clinical trials designs for oncology treatment

The way in which cancer is treated has moved from non-specific cytotoxic chemotherapy - which kills both cancerous and normal cells that divide rapidly - to the more targeted treatments. Targeted therapies improve selectivity of drug delivery while providing less adverse effects. Traditional cytotoxic medications primarily work by damaging DNA and other rapidly dividing cells. The therapeutic dose is determined during Phase I clinical trials based on the maximum tolerated dose (Fox et al., 2002). Tumour response is generally evaluated by assessing the extent of tumour shrinkage.

Targeted therapies focus specifically on proteins, receptors and/or signalling pathways which contribute to malignant transformation, death of the cell through apoptosis and/or cell cycle regulation (Verweij et al., 2019). Targeted therapies are usually developed for specific cancers that have distinct molecular changes, thus there may be fewer patients involved in biomarker-directed trials of targeted drugs compared to those involved in traditional drug trials. Larotrectinib was tested for several different types of tumours with approved molecular targets, but very few patients were included for this type of trial (Goldberg et al., 2017).

There are four phases in an oncology drug clinical trial: phase 1 (effects of dosage and toxicity); phase 2 (evidence of some benefit); phase 3 (comparison of the investigational agent against some standard treatment in large numbers of people); and phase 4 (monitoring of the drug following approval for post-marketing safety). Evidence shows that targeted therapy has improved outcomes achieved with chemotherapy (e.g., gained median survival with colorectal cancer) in addition to those gained through chemotherapy alone.

Therefore, there are some studies using the Bayesian approach, such as the I-SPY breast cancer trials that were conducted using this type of adaptive design, that have correctly identified potentially promising agents for further assessment as a result of their ability to analyze interim results (Goldberg et al., 2017).

**FIGURE 10:**
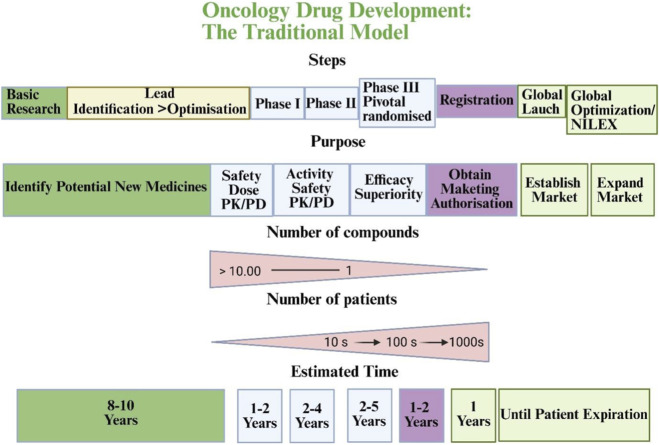
The traditional rocket model for oncology drug development (Verweij et al., 2019).

Traditional cancer clinical trials have serious problems, such as high costs and long times for completion, as well as drop-outs from participants, especially in Phase III, which can impact the result (Verweij et al., 2019) (Figure 10). In addition, many cancers that look the same under a microscope are actually different at the molecular level, so there probably will not be one standard treatment for all patients with a particular type of cancer (Kaplan et al., 2013). Because of this, we need new designs for trials that can more quickly and less expensively evaluate new drugs across defined molecular subgroups (Goldberg et al., 2017).

Biomarker-based trial designs will help discover new targeted treatments for cancer. An example includes evaluations through retrospective analysis of previously completed trials. For instance, KRAS mutations can be predictive biomarkers of the response to EGFR inhibitors for the treatment of colorectal cancer; these predictive biomarkers will then be confirmed through future prospective studies. In population-enriched designs, only patients who have specific biomarkers (e.g., HER2 amplification for patients with breast cancer and ALK rearrangements for patients with lung cancer) will be enrolled. Although using this design is likely to yield great results, identifying the specific biomarkers may add significant expense (particularly when the biomarkers of interest are non-typical examples) to the screening process (Kaplan et al., 2013).

Biomarker-stratified trial designs assess the effect of both treatment and biomarker in a single trial design; however, this typically requires large sample sizes. In addition, single-arm trials may be done when necessary to evaluate rare cancers, so that the trial results may be compared to historical controls (Goldberg et al., 2017).

Most recently, adaptive trial designs and multi-arm trial designs such as I-SPY and BATTLE have been created as ways to evaluate several treatments or biomarkers simultaneously. While these designs typically add value through increased efficiency and reduced cost, they also tend to be more difficult to operationalize and often come with higher probabilities of statistical error. Regardless, having innovative trial designs is crucial for advancing precision oncology in an era of molecularly targeted therapies.

The following are innovative types of clinical trials applied to accelerate the development of anticancer drugs:

### 12.1 Basket trials

The basket trial concept is involved in using a specific cancer therapy for multiple different cancers based on their shared genetic mutation without regard to their tissue type (Sosman et al., 2012). The efficacy of the cancer therapy is judged by whether the agent demonstrates an ability to inhibit the specific mutation and suppress the growth of cancer. Patients with rare cancers or malignancies stand to benefit greatly from the use of basket trials as they provide an accelerated approach for assessing new therapies across multiple cancer types (Kopetz et al., 2015).

Nevertheless, a common alteration does not necessarily cause tumour development; there are also tumour-specific biological characteristics that may affect treatment response (Hyman et al., 2015). One example of this phenomenon is seen with vemurafenib, which has demonstrated efficacy in melanoma with BRAF V600 mutations but limits efficacy in colorectal cancer with the same BRAF mutation because of tumour-specific resistance mechanisms. Phase II basket trials and NCI-MATCH (Abrams et al., 2014) have utilized this model to evaluate target therapies based upon the molecular alterations of the patient grouped as opposed to the histopathology.

**FIGURE 11:**
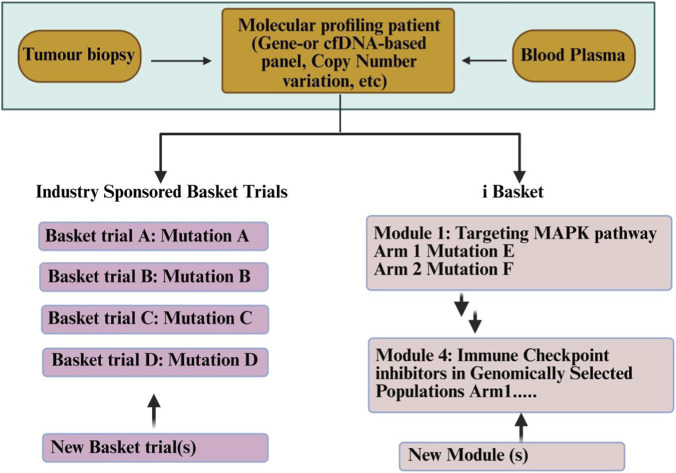
Design of different types of basket trials (Verweij et al., 2019).

Rare cancers are defined as those that occur in six or fewer persons annually per 100,000. Rare cancers now comprise more than 20% of the total number of cancers diagnosed, and an increasing percentage of rare cancers are being classified based upon their molecular biomarkers as compared to their histological characteristics. To summarize, basket trials are an important advancement in precision oncology, allowing for the development of drugs based upon biomarkers and across multiple tumour types (Billingham et al., 2016). Figure 11 illustrates different designs of Basket Trials developed by the Cancer Core Europe (Verweij et al., 2019).

### 12.2 Umbrella trials

Umbrella clinical trials assess multiple targeted therapies for a specific type of cancer (same histology) in patients with genetically diverse subgroups. Patients are grouped based on their biomarkers, and each group then received a unique treatment, which can then be compared to a standard treatment in a randomised sub-study. As a result, researchers can compare how patients respond to drugs when there are several genetically different types of tumours, yet challenges remain in being able to recruit sufficient numbers of patients who have rare mutations (Gandara et al., 2017). The FOCUS4 trial in colorectal cancer provides an excellent illustration of this concept: patients were screened for biomarkers at the initiation of their chemotherapy and then received targeted therapies based on their molecular profiles.” The BFAST study evaluated atezolizumab for patients with advanced NSCLC, and also used mutations to separate patients to evaluate the differences in biomarker-based response to therapy. Other umbrella trials have similarly incorporated biomarker screening into the selection of targeted therapies for patients with lung cancer (Steuer et al., 2015). Overall, umbrella trials provide an additional avenue for making targeted therapies more precise and helping to identify matched therapies for the same tumour type according to their molecular subtype.

Rare cancers or situations where a new treatment has demonstrated clear success use historical control studies as a method of trialing new therapies. These single-arm studies compare average results from new patients treated with the investigational drug to previously conducted trials. While these types of trials can be done in practice, they will not typically have randomization and blinding which will provide an increased susceptibility to bias and confounding variables (Verweij et al., 2019).

Phase I expansion cohort trials begin with determining safety, maximum tolerated dose, and pharmacokinetics; then, these trials expand the number of patients in a cohort to evaluate potential biomarker performance, potential medication interactions such as drug-drug, monotherapy performance, or combination performance. This flexible approach will facilitate an initial indication of efficacy as it allows a trial to identify efficacy signals before any additional trials are launched.

Parallel-group trials may be designed with a common control arm to evaluate multiple new medications against a common control group, thus minimizing sample size and resource use which makes this trial design advantageous in rare cancers. Arms may open at varying times in order to allow for overlap in enrollment periods (Goldberg et al., 2017).

Proper definition of endpoints for clinical trials is also difficult as response criteria are dependent on tumour type, site, and symptom burden. Endpoint considerations should also include the depth, duration, and clinical relevance of responses. Successful trials rely on agreement from the clinicians, statisticians, and developer’s side in order to achieve an appropriate balance between meaningful efficacy and the statistical validity required to meet regulatory approval threshold.

## 13 Future perspectives and innovations

Continued progress in cancer research necessitates the development of more sophisticated, biologically relevant, and translationally predictive model systems. The integration of cutting-edge technologies such as multi-omics approaches, 4D bioprinting, artificial intelligence (AI), and patient-derived organoids offers transformative potential to address the inherent limitations of traditional *in vitro* and *in vivo* models, thereby accelerating the discovery and optimization of personalized cancer therapies (Debela et al., 2021).

### 13.1 Integration of multi-omics with cancer models

The integration of multiple omics technologies will lead to improvements in understanding tumor biology through the use of both *in vitro* and *in vivo* models (Correa-Aguila et al., 2022). In addition, these multi-omic approaches will provide a comprehensive molecular understanding of how tumors initiate, grow, and respond to treatment. Multi-omic studies performed through use of either organoids or patient-derived xenograft (PDX) models are assisting in identifying major drivers, resistance mechanisms, and predictive biomarkers, facilitating personalized treatment options (Subramanian et al., 2020), with ongoing initiatives focusing on implementing real-time monitoring of the omic data generated, and, as science continues to unfold for new ways in which to integrate the data using standardized formats, enhancing the ability to translate the data into clinically useful applications (Pettini et al., 2021).

### 13.2 Development of 4D bioprinting

Using 4D bioprinting to create *in vitro* tumour constructs, researchers are able to create tissue mimetics that can adaptively respond to chemical and mechanical stimuli from their environment and therapies (Shukla et al., 2024). By using stimuli-responsive biomaterials and bio inks with both cancerous and non-cancerous/stromal cells, these models create more physiologically-relevant representations of tumour growth, angiogenesis, and metastasis than traditional 2D *in vitro* cell cultures or scaffolds (Khorsandi et al., 2024). This adaptive nature will also allow for more accurate studies on time-dependent drug responses (Jung et al., 2022).

### 13.3 AI-driven modelling and predictive analytics

AI and ML are now used to analyze large datasets from organoid models, PDX models, and imaging platforms, detecting predictive markers, estimating responses to therapy, and developing combination therapies (Alum, 2025). AI-driven automation assists in enhancing both the efficiency and reproducibility of high-throughput research through the use of Deep Learning models for simulating tumour progression and providing a basis for virtual drug screening (Huhulea et al., 2025).

### 13.4 Expansion of patient-derived organoids (PDOs)

PDOs (Patient-derived Organoids) are 3D tumor models produced from patients’ individual tissues which maintain the original tumor’s histological and genetic characteristics. PIDOs are available in large numbers and can be used on drug screening platforms This makes PDOs very good for the testing of customized treatments and for use towards cancer research in the translational aspect (Granat et al., 2019; Pamarthy and Sabaawy, 2021; Wu et al., 2022).

## 14 Future directions and innovations in PDO technology

Future innovations in patient-derived organoid (PDO) research are focused on enhancing their physiological and translational relevance. Co-culturing PDOs with immune cells, fibroblasts, or endothelial components enables a more accurate reconstruction of the tumour microenvironment, capturing critical cellular interactions that drive cancer progression and therapy resistance. The integration of single-cell sequencing and CRISPR-based functional screening further allows the dissection of molecular heterogeneity and identification of resistance mechanisms, supporting the development of precision oncology strategies. Moreover, the establishment of living biobanks comprising organoids from diverse cancer subtypes and patient populations represents a major step toward personalized clinical decision-making and drug discovery pipelines (Joshi et al., 2020).

### 14.1 Clinical translation and FDA-approved therapies

From fundamental discoveries at the molecular level to more advanced, targeted therapies based on individual characteristics, cancer research has progressed over time. The combination of experimental models (typically performed in a laboratory), genomic profiling, and bioinformatics with high throughput screening for drug discovery has led to the accelerated development and approval of new targeted agents, such as those used in immunotherapy, antibody drug conjugates, PARP inhibitors, and tumor-agnostic agents. This proves that translational research is an essential part of moving research out of the lab and into the clinic (Scott et al., 2023; Biersack and Höpfner, 2024).

#### 14.1.1 Targeted therapies

Targeted cancer therapies block multiple, specific, molecular causes of cancer from causing cancer. One example is imatinib, which blocks BCR-ABL—the fusion protein found in patients with chronic myeloid leukaemia. Erlotinib and gefitinib block EGFR mutations found in non-small cell lung cancer, and trastuzumab blocks HER2-positive breast cancer (de Martel et al., 2020; Debela et al., 2021). These examples illustrate how molecular profiling allows for precision-medicine approaches to treating cancer (Lee et al., 2018; Choi and Chang, 2023; Zhong et al., 2021).

#### 14.1.2 Immunotherapies and checkpoint inhibitors

Immune checkpoint inhibitors, including Nivolumab and Pembrolizumab, block signalling of PD-1, thus improving T-cell response in many cancers (Shiravand et al., 2022). Ipilimumab targets CTLA-4 in metastatic melanoma. CAR-T therapies, such as Axicabtagene ciloleucel and Tisagenlecleucel, utilise genetically engineered T cells to treat specific haematological malignancies, yielding high and previously unreached outcomes (Sharpe, 2017; Naimi et al., 2022).

#### 14.1.3 Antibody–Drug Conjugates (ADCs)

Monoclonal antibodies target specific tumor antigens in combination with highly potent cytotoxic medicines that are delivered directly to malignant cells via an antibody–drug conjugate (Marei et al., 2022). Examples of approved antibody–drug conjugates include Brentuximab vedotin (Hodgkin lymphoma), Trastuzumab emtansine (HER2 positive breast cancer), and Enfortumab vedotin (urothelial carcinoma). Antibody–drug conjugates are effective treatments with reduced toxicity to normal tissues (Drago et al., 2021).

#### 14.1.4 PARP inhibitors and synthetic lethality

Poly (ADP-ribose) polymerase (PARP) inhibitors take advantage of the defective DNA repair systems found in some cancers, particularly those with mutations in the BRCA gene (Li et al., 2022). Examples of approved PARP inhibitors include Olaparib, Rucaparib, Niraparib, and Talazoparib. All four of these agents kill tumor cells through synthetic lethality due to their homologous recombination deficiency. The development of PARP inhibitors is one of the best examples of how biomarker-driven therapies can successfully treat cancer (Yang et al., 2019).

#### 14.1.5 Tumour-agnostic therapies

Tumor-agnostic therapies have been authorized based on particular genetic changes rather than the place of the patient’s tumor where it originated (Chandrasekaran and Elias, 2021). Examples of this include Larotrectinib and Entrectinib which treat solid tumors that have NTRK gene fusions regardless of where they developed and Dostarlimab which is given to every patient with mismatch repair-deficient (dMMR) or methylated tumors. Overall, the development of these therapies has changed the way in which we view oncology as moving from tissue-based therapies to those that are determined through genomic data (Weymann et al., 2023).

#### 14.1.6 Companion diagnostics and biomarker-based approaches

Personalized cancer therapy uses companion diagnostics (testing) to allow patients to receive therapies that match their cancer type or specific mutations within the cancer. For instance, to administer an EGFR inhibitor, you must first determine if the patient has a mutation in the EGFR gene, then according to the PD-L1 assay results, you can determine if your patient will respond to a checkpoint inhibitor, and finally, the same is true for patients who require a PARP inhibitor (BRCA testing). New technologies (i.e., organoids) and more sophisticated *in vitro* testing methods are helping to provide evidence to better support these biomarkers and create individualized treatment plans faster (Orellana García et al., 2021; Chirmule and Ghalsasi, 2025).

### 14.2 Roles of biomolecules in cancer

Cancer progression results from complex alterations in biomolecules that regulate cellular homeostasis.

#### 14.2.1 DNA and RNA

Gene mutations, copy-number changes, gene fusions and epigenetic modifications are thought to initiate the development of cancer (tumourigenesis). The modification of RNA, such as abnormal splicing and changes in the expression of non-coding RNAs, furthers the development of oncogenic signalling and the progression of tumours (Hanahan and Weinberg, 2011; Esteller, 2011; Rupaimoole and Slack, 2017).

#### 14.2.2 Proteins

Aberrant activation of receptor tyrosine kinases and downstream pathways such as PI3K–AKT–mTOR drives proliferation and survival (Zhao et al., 2021). Imbalances in apoptosis-regulating proteins and proteases contribute to resistance and metastasis (Dang, 2012; Zhao et al., 2021).

#### 14.2.3 Lipids

Tumour cells have adapted to increase their capacity for the *de novo* synthesis of lipids and lipids signalling pathways to aid in the construction of new membranes, the storage of energy, and the modulation of the immune response (Menendez and Lupu, 2007). Changes in the lipid content can also contribute to the development of resistance to drug therapies (Beloribi-Djefaflia et al., 2016).

#### 14.2.4 Carbohydrates

Abnormal glycosylation patterns aid in a tumour’s ability to evade the immune system, invade and spread to metastatic sites through alterations in cell–cell and cell–matrix interactions (Pinho and Reis, 2015; Pearce and Läubli, 2016).

#### 14.2.5 Metabolites

Metabolic reprogramming, including the Warburg effect (aerobic glycolysis), not only supports the rapid growth of cancer cells, but connects the processes of metabolism to the regulation of epigenetic processes (Vander Heiden et al., 2009; Pavlova and Thompson, 2016).

#### 14.26 Extracellular vesicles

Cancer-derived vesicles transfer oncogenic molecules, remodel the tumour microenvironment, and promote drug resistance (Wortzel et al., 2019; Kalluri and LeBleu, 2020).

#### 14.27 Post-translational modifications

Dysfunctional phosphorylation, ubiquitination, and other modifications lead to changes in protein stability and function which enhance the signaling pathways of cancer cells (Zhao et al., 2021; Banani et al., 2017).

## 15 Integrated perspective

Together, the changes in nucleic acids, proteins, lipids, carbohydrates, metabolites and extracellular components produce malignant phenotypes.

### 15.1 Molecular mechanism of biomolecular phase separation

**FIGURE 12:**
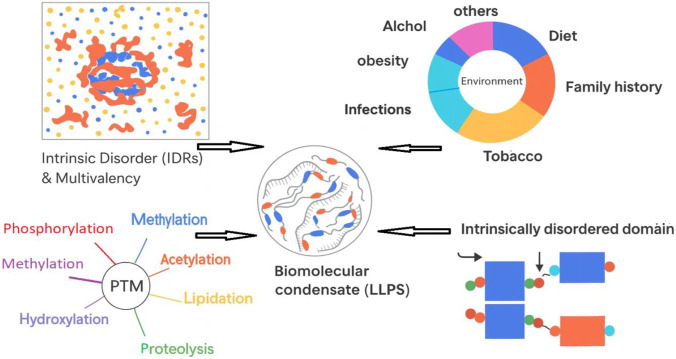
Drivers and regulators of LLPS. Intrinsically disordered regions (IDRs), multivalent domains, RNA scaffolds, post-translational modifications, and concentration changes.

Liquid–liquid phase separation (LLPS) is a physicochemical (Yang et al., 2019; Yin et al., 2014) process in which a homogeneous solution of biomolecules separates into two distinct coexisting phases: a dense phase enriched in proteins and/or RNAs (the condensate) and a surrounding, dilute phase. Cells employ LLPS to assemble membrane less compartments, such as nucleoli, stress granules, P-bodies, and transcriptional condensates. These dynamic compartments enable the spatial and temporal regulation of biochemical reactions by concentrating selected components, excluding inhibitors, and buffering fluctuations in molecular concentration (Banani et al., 2017) (Figure 12).

Sequence-encoded drivers: intrinsically disordered regions and the stickers-and-spacers model. The key molecular drivers of LLPS are intrinsically disordered regions (IDRs) and low-complexity domains (LCDs). Unlike folded domains, IDRs lack a stable tertiary structure and mediate multiple weak transient interactions. Conceptual frameworks describe these regions in terms of “stickers” (residues or motifs that mediate transient associative contacts, such as aromatic residues or arginine-rich motifs) and “spacers” (segments that modulate solubility and set the distance between stickers). The stickers-and-spacers model explains how valence, sticker chemistry, and patterning determine both the saturation concentration for phase separation and the material properties of condensates, ranging from liquid-to gel-like states (Shin et al., 2017; Bremer et al., 2022).

#### 15.1.1 Role of RNA and multicomponent interactions

Evidence now shows that RNA functions to actively regulate biomolecular condensates instead of acting merely as a passive cargo. The binding of RNA to different RNA-binding proteins depends on the length, sequence motifs, structure and chemical modifications (e.g., m6A) present on the RNA molecule. At low ratios of RNA:protein, RNA promotes phase separation by providing a multivalent scaffold for the formation of the condensate, however, once the RNA:molecule ratio exceeds a certain threshold, excess RNA will result in the dissolution of the condensate (reentrant behaviour). The interactions between proteins and/or RNAs give rise to the formation of complex, multilayered condensates that have an intrinsic internal organisation (Roden and Gladfelter, 2021).

## 16 Physical chemistry and phase behavior

Pathways associated with phase separation parallel classical thermodynamic principles associated with multicomponent systems. Formation of a condensate occurs once an individual biomolecule has reached a concentration beyond a critical value within the two-phase region identified by the binodal and spinodal boundaries (Dignon et al., 2018). Formation of a condensate is influenced by several factors; temperature, pH, ionic strength, concentration and strength of interaction all contribute to determine whether a condensate forms. Increasing the valency of the molecules or increasing the strength of the interaction motifs will decrease the saturation threshold required for the condensation of biomolecules (Hyman et al., 2014; Maharana et al., 2018).

### 16.1 Regulation mechanisms

Cells use a variety of different ways to control their condensates through post-translational modifications (MOD), including using various methods to increase or decrease the charge and/or strength of their interactions with one another via phosphorylation, methylation and acetylation, ubiquitination, etc. In addition, cells can use ATP-driven molecular chaperones to help prevent irreversible aggregation of condensates. Based on studies using synthetic systems (such as optogenetic tools) to understand how condensates form, it is clear that making changes in valency or recruitment amounts to sufficient triggering of condensate formation, while excessively stabilising them can induce their maturation into non-dynamic forms (Alberti et al., 2019).

### 16.2 Material properties and pathological transitions

Condensates exhibit dynamic clothing liquid behaviours, such as fusing together and exchanging molecules with one another. Over a period of time or during periods of high stress, they typically transition to gelled or amyloid-like aggregate forms. In pathological conditions, many disease-causing mutations lead to an increase in the strength of interactions, thereby reducing the threshold for abnormally solidifying. As such, the outcome of those abnormally changing behaviours has been observed in both neurodegenerative and cancer-associated diseases, as altered physical states of material significantly interfere with regular cellular processes (Hyman et al., 2014; Banani et al., 2017).

### 16.3 Functional consequences in cells

Biochemical reactions can be organized when specific molecules are concentrated in space via condensation. Some examples of condensates in cells are nucleoli (ribosome assembly), stress granules, P-bodies, and transcriptional condensates at super-enhancers, all of which create pathways in which molecules undergo biochemical reactions. When the condensation process is dysregulated, there is potential for oncogenic transcription, decreased efficiency of DNA repair, and alteration of immune signaling, thus providing a new class of targets for therapeutics.

## 17 Conclusion

In modern oncological medicine advances in molecular profiling, improved experimental animal models, and adaptive clinical trial designs are converging in ways that have dramatically changed how oncologists approach the treatment of cancer. Previously, the use of genomic, transcriptomic, and functional data from *in vitro* cell lines and other preclinical models allowed oncologists to accurately predict whether a targeted new drug would work against specific cancers. This convergence of technologies has accelerated the advent of precision medicine through the accurate prediction of therapeutic efficacy using target agents (MEK, PI3K, and FGFR inhibitors) (Banani et al., 2017; Varki, 2017).

Cancer cell lines continue to be vital resources in clinical cancer research for three primary reasons: they are reproducible; they can be scaled to allow for the generation of sufficient quantities for both preclinical and clinical trials; and they provide a translational link from basic laboratory research to clinical use.

Emerging technologies, such as multi-omics integration, artificial intelligence (AI)-driven analytics, and physiologically relevant model systems, are providing novel ways to refine drug development pipelines and enhance biomarker-guided therapeutic strategies. In addition, natural product research offers an additional avenue for innovation. There are many plants that have been identified to have anti-cancer properties through the modulation of oncogenic signaling pathway(s); however, only a very small proportion of the estimated number of plant phytochemicals have been thoroughly studied. Expanding the phytochemical and mechanistic investigations of less-studied plant species will ultimately identify previously unknown bioactive compounds, or candidate therapeutic scaffolds.

In summary, ongoing integration of rigorous preclinical modeling, molecular stratification, novel trial methodologies, and systematic exploration of natural bioresources are necessary to develop personalized, safe, and globally accessible therapies for cancer.

## Glossary

P13K/AKT: AKT/mTOR/phosphoinositol-3-kinase
MAPK: mitogen-activated
ICD: International Statistical Classification of Diseases
WHO: World Health Organization’s
DALYs: disability-adjusted life years
GBD: Global Burden of Disease
CFR: case fatality rate
GLOBOCAN: Global Cancer Observatory
UV: ultraviolet
BMI: body mass index
RCC: VHL: von Hippel–Lindau
HIF: hypoxia-inducible factor
HNSCC: Head and neck squamous cell carcinoma
EGFR: epidermal growth factor receptor
NSCLC: non-small cell lung cancer
MPM: malignant pleural mesothelioma
PDAC: pancreatic ductal adenocarcinoma
EMT: epithelial–mesenchymal transition
PTC: papillary thyroid carcinoma
ATC: anaplastic thyroid carcinoma
FTC: follicular thyroid carcinoma
SCLC: Small cell lung cancer
NHL: non-Hodgkin lymphoma
EBV: Epstein–Barr virus
DLBCL: diffuse large B-cell lymphoma
HCC: hepatocellular carcinoma
HCV: hepatitis C virus
AFP: alpha-fetoprotein
HBV: hepatitis B virus
EGCG: Epigallocatechin gallate
CTCs: circulating tumour cells
ctDNA: circulating tumour DNA
AI: Artificial intelligence
PDT: Photodynamic therapy
ROS: reactive oxygen species
ADCs: antibody–drug conjugates
API: active pharmaceutical ingredient
DAR: drug-to-antibody ratio
PD-1: Programmed cell death protein 1
NK: natural killer
mAbs: Monoclonal antibodies
SG: sacituzumab govitecan
TDXd: fam-trastuzumab deruxtecan
T-DM1: trastuzumab emtansine
NSCC: Non-Small Cell Carcinoma
ALK: anaplastic lymphoma kinase
BATTLE: Biomarker Integrated Approaches of Targeted Therapy for Lung Cancer Elimination
NCI MATCH: National Institute of Cancer Match Trial
BFAST: Blood First Assay Screening Trial
SCC: squamous cell carcinoma
PDX: patient-derived xenograft
ML: machine learning
PDOs: Patient-Derived Organoids
3D: three-dimensional
TKI: tyrosine kinase inhibitor
CML: chronic myeloid leukaemia
HER2: human epidermal growth factor receptor 2
dMMR: Dostarlimab–for mismatch repair-deficient
CDx: companion diagnostics
LLPS: Liquid–liquid phase separation
IDRs: intrinsically disordered regions
LCDs: low-complexity domains
PTMs: post-translational modifications
MEK: MAPK/ERK kinase
P13K: phosphoinositide-3-kinase
FGFR: fibroblast growth factor receptor
